# Iron oxide nanoparticles empowering melanoma therapy: advances in multifunctional platform research

**DOI:** 10.3389/fbioe.2025.1655677

**Published:** 2025-11-07

**Authors:** Xiaolong Bai, Zhijun Wang, Ziyi Cao, Junping Zhen

**Affiliations:** 1 College of Medical Imaging, Shanxi Medical University, Taiyuan, Shanxi, China; 2 Department of Imaging, Second Hospital of Shanxi Medical University, Taiyuan, Shanxi, China; 3 Second Clinical Medical College, Shanxi Medical University, Taiyuan, Shanxi, China; 4 First Clinical Medical College, Shanxi Medical University, Taiyuan, Shanxi, China; 5 Molecular Imaging Laboratory, Second Hospital of Shanxi Medical University, Taiyuan, Shanxi, China

**Keywords:** melanoma, iron oxide nanoparticles, photodynamic therapy, immunotherapy, nanodrug delivery

## Abstract

Melanoma, one of the most aggressive types of skin cancer, has exhibited a continuously rising incidence worldwide, accompanied by a significant mortality risk. Despite ongoing advances in therapeutic approaches, effective and definitive clinical interventions remain limited, severely restricting long-term patient prognosis. In recent years, nanotechnology-based tumor therapies have demonstrated tremendous potential. Among them, iron oxide nanoparticles (IONPs), owing to their excellent biocompatibility, magnetic responsiveness, low cost, and scalability in synthesis, have emerged as a highly promising nanoplatform for melanoma treatment. This review systematically summarizes the physicochemical properties of IONPs and their tumor-targeting mechanisms, with a particular focus on recent advances in their applications for melanoma, including drug-targeted delivery, hyperthermia, Photodynamic therapy (PDT), Chemodynamic therapy (CDT), immunotherapy, and combination therapies. Additionally, the review discusses current challenges and provides perspectives on the future development of IONPs for precision melanoma therapy.

## 1 Introduction

Skin cancer is one of the most common malignancies worldwide, and its incidence has been steadily increasing in recent years. Based on histological origin, skin cancers are primarily categorized into non-melanoma skin cancers and melanoma (Liu-Smith et al., 2017; Apalla et al., 2017). Although melanoma accounts for only approximately 2% of all skin cancer cases, it is responsible for nearly 75% of skin cancer-related deaths. Its aggressive nature and high metastatic potential render it a major public health concern. Particularly among individuals with fair skin, the incidence of melanoma is rising at an annual rate of 4%–6% (Gray-Schopfer et al., 2007; Shadbad et al., 2021). Melanoma originates from melanocytes located in the basal layer of the epidermis. These cells are not only responsible for melanin synthesis but also play a critical role in protecting the skin from ultraviolet (UV) radiation-induced damage. The pathogenesis of melanoma is complex and multifactorial, influenced by genetic susceptibility, UV exposure, immunosuppressive states, family history, and environmental factors (Switzer et al., 2022; Gladfelter et al., 2017). With advances in our understanding of its molecular and pathological mechanisms, melanoma is now clinically staged from I to IV, with stage IV—referred to as metastatic melanoma—being particularly difficult to treat and associated with a poor prognosis, with a 5-year survival rate ranging from only 5%–19% (Naidoo et al., 2018; Nguyen et al., 2020). Current treatment modalities for melanoma include surgical excision, radiotherapy, chemotherapy, immunotherapy, and targeted therapy. While surgical resection is often effective in early-stage melanoma, conventional therapies offer limited efficacy for advanced or metastatic disease and are frequently accompanied by significant adverse effects (Domingues et al., 2018; Tang et al., 2017). In recent years, immune checkpoint inhibitors (e.g., anti-PD-1 and anti-CTLA-4 antibodies) and targeted therapies against BRAF mutations have significantly improved clinical outcomes in selected patient populations. However, the development of drug resistance and immune-related adverse events continues to pose major therapeutic challenges (Dhillon et al., 2020). Consequently, there is an urgent need to develop more effective, safer, and controllable therapeutic strategies for melanoma management.

**FIGURE 1:**
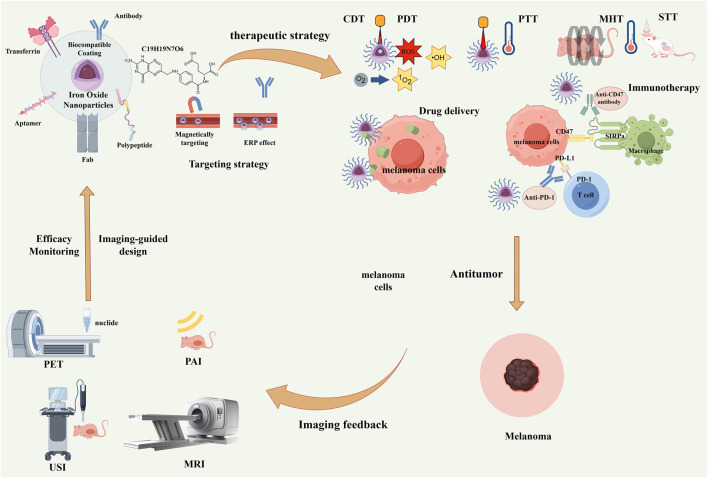
Schematic illustration of multifunctional iron oxide nanoparticles (IONPs) for integrated diagnosis and therapy of melanoma. Through functionalized surface modification with targeting ligands such as antibodies and peptides, combined with magnetic targeting and the enhanced permeability and retention (EPR) effect, precise tumor targeting can be achieved. Subsequently, multiple therapeutic modalities—including chemodynamic therapy (CDT), photodynamic therapy (PDT), photothermal therapy (PTT), magnetic hyperthermia therapy (MHT), sonodynamic therapy (STT), drug delivery, and immunotherapy—can be implemented. Meanwhile, multimodal imaging techniques such as PET, PAI, USI, and MRI enable real-time therapeutic monitoring and imaging feedback, ultimately forming a complete “imaging–therapy–imaging feedback” theranostic loop.

With the rapid advancement of nanotechnology, inorganic nanomaterials have attracted increasing attention due to their potential applications in cancer therapy and molecular imaging. These materials have been widely explored in various biomedical fields, including biosensing, bioimaging, targeted drug delivery, as well as antibacterial and antitumor therapies (Duan et al., 2024). However, among inorganic nanomedicines, only iron-based nanomaterials have been approved by the U.S. Food and Drug Administration (FDA). Nanomaterials are typically defined as structures with at least one dimension less than 100 nm. To prevent rapid renal clearance, the particle diameter should generally be no less than 10 nm. In addition, to avoid vascular embolism or nonspecific phagocytosis by the reticuloendothelial system (RES), the maximum size should not exceed 180–200 nm (Chomoucka et al., 2010). Recent studies have shown that, compared with other types of nanoparticles, iron oxide nanoparticles (IONPs) exhibit excellent biocompatibility, strong magnetic responsiveness, and ease of surface modification and functionalization (Yang et al., 2023; Singh et al., 2025) (Table 1). As non-invasive molecular imaging probes, IONPs have been increasingly employed in emerging strategies for the diagnosis and treatment of melanoma (Chen F. et al., 2023; Cruz et al., 2020; Marzi et al., 2022) (Figure 1). Despite this growing interest, comprehensive and systematic reviews on the use of IONPs in melanoma therapy remain scarce. Therefore, this review focuses on the potential applications of IONPs in melanoma, highlighting their key physicochemical properties and tumor-targeting mechanisms. Furthermore, it summarizes the current progress in various therapeutic strategies involving IONPs and discusses future directions, aiming to provide valuable insights for further research and clinical translation in this field.

**TABLE 1:** Comparison of the characteristics and therapeutic outcomes of various types of nanocarriers commonly used in melanoma treatment.

Nanocarrier type	Main composition/Structural characteristics	Advantages	Disadvantages	Reported therapeutic outcomes	Key references
Iron oxide nanoparticles (IONPs)	Fe_3_O_4_ or γ-Fe_2_O_3_ core, surface coated with PEG, chitosan, or lipid layers	Excellent magnetic responsiveness and imaging visibility, enabling magnetically guided drug delivery and localized hyperthermia; possesses theranostic potential; good biodegradability	Complex surface modification and functionalization process; limited control over particle size and colloidal stability; high doses may induce ROS accumulation and cellular stress, raising concerns about long-term safety	Significantly inhibited B16-F10 melanoma growth (tumor inhibition rate 60%–80%); combined magnetic hyperthermia markedly prolonged mouse survival	Patrick et al. (2024)
Liposomes	Phospholipid bilayer vesicular structure capable of encapsulating both hydrophilic and hydrophobic drugs	Excellent biocompatibility and membrane fusion capability; mature preparation technology; some formulations already approved for clinical use (e.g., Doxil®)	Limited stability with potential for drug leakage or fusion degradation; short circulation time; targeting efficiency mainly relies on the EPR effect, leading to passive targeting limitations	Moderate tumor inhibition (40%–60%) in melanoma models; often used in combined chemotherapy and immunotherapy strategies	Corte-Real et al. (2024)
Polymeric nanoparticles (PLGA, PEG-PLGA, etc.)	Biodegradable polymeric backbone with stable structure	Controlled drug release profiles, high stability in blood, low immunogenicity; tunable fabrication parameters suitable for sustained-release systems	Complex synthesis procedures affected by solvent conditions and polymer molecular weight; depend primarily on the EPR effect for passive targeting; active-targeting efficiency remains limited; degradation products (lactic and glycolic acid) may cause local acidification and drug inactivation	Significantly extended plasma half-life and doubled pharmacological efficacy; low *in vivo* toxicity	Janaszewska et al. (2015)
Copper nanoparticles (CuNPs)	Metallic copper core at the nanoscale, capable of forming stable composites with organic molecules or polymers	Induce ROS generation via Fenton-like reactions for selective tumor cell killing and mitochondrial pathway activation; possess dual mechanisms of chemotherapy and oxidative stress induction	Prone to oxidation and aggregation; poor stability and controlled release; may cause nonspecific oxidative damage and systemic toxicity *in vivo*	Induced caspase-3 activation, mitochondrial membrane potential loss, and cytochrome c release in B16F10 melanoma cells; demonstrated significant apoptosis induction	Pojo et al. (2013)
Gold nanoparticles (AuNPs)	Gold core with PEG or functional peptide surface modification	Excellent photothermal conversion efficiency and imaging capability; precise light-responsive control enabling visualized hyperthermia and synergistic drug release	High cost; uncertain long-term biosafety and metabolic pathways *in vivo*; strongly dependent on laser parameters	Tumor inhibition rate up to 90% under laser irradiation; demonstrated synergistic therapeutic enhancement in combination therapy	Vines et al. (2019)
Carbon-based nanomaterials (Graphene Oxide, CNTs)	Carbon backbone with ultrahigh specific surface area	High drug-loading capacity and excellent photothermal properties; suitable for NIR photothermal–chemotherapy combination; good mechanical stability	Poor biodegradability; potential cytotoxicity and immune response risks; difficult *in vivo* clearance and metabolism	Combined photothermal therapy and drug delivery significantly inhibited melanoma tumor growth in mouse models	de Carvalho Lima et al. (2022)
IONP-based Hybrid Nanoplatforms for Combined Therapy	Core–shell or multilayer hybrid structures integrating magnetic responsiveness with high drug-loading capacity	Integrate magnetic targeting, imaging monitoring, and controlled drug release; achieve theranostic functionality; markedly improve drug delivery efficiency	Complex fabrication processes require external magnetic field equipment; reproducibility and large-scale production need further optimization	*In vivo* tumor growth suppression >85%, demonstrating excellent precision therapeutic potential	García-Soriano et al. (2024)

## 2 The properties of IONPs

IONPs are generally composed of crystalline structures such as hematite (α-Fe_2_O_3_), magnetite (Fe_3_O_4_), or maghemite (γ-Fe_2_O_3_). These materials exhibit favorable biocompatibility, gradual biodegradability under physiological conditions, and low systemic toxicity (Liu et al., 2025; Daya et al., 2022). In particular, ferromagnetic or ferrimagnetic nanomaterials can display superparamagnetic behavior under specific conditions. When exposed to an external weak magnetic field, they respond rapidly and can be effectively guided toward target sites; once the field is removed, their magnetization vanishes promptly, thereby avoiding residual magnetism *in vivo* (Li et al., 2013; Gao et al., 2009). Duan et al. (2022) investigated the potential effects of electromagnetic fields on melanoma progression and demonstrated that such fields not only modulate the biological behavior of melanoma cells but also significantly enhance the uptake efficiency of anticancer drugs and therapeutic outcomes. For instance, static magnetic fields can be utilized to guide IONPs precisely to the tumor site, enabling site-specific and on-demand drug delivery. Alternating magnetic field (AMF) can activate magnetic nanoparticles embedded within thermosensitive polymer matrices, inducing local hyperthermia through magnetic-to-thermal energy conversion (magnetothermal effect), thereby triggering controlled drug release at the tumor site (Hu et al., 2007). Studies have further shown that IONPs with smaller sizes and optimized morphologies possess enhanced magnetic relaxivity and reduced toxicity, facilitating more efficient inhibition of tumor cells (Malhotra et al., 2020). Paez-Muñoz et al. (2023) compared spherical and cubic magnetic nanoparticles with similar magnetic and hyperthermic properties. In tumor-bearing mice, spherical nanoparticles demonstrated higher magnetic relaxivity and lower cytotoxicity than their cubic counterparts, while both exhibited comparable specific absorption rates (SAR). In contrast, other studies reported that rod-shaped iron oxide nanoparticles are more cytotoxic than spherical ones (Hu et al., 2007). These findings indicate that the cytotoxic potential of nanoparticles should be evaluated based on their shape and size, as these parameters directly influence surface area, which is closely associated with cellular toxicity (Kladko et al., 2021).

However, IONPs still face stability challenges in aqueous or physiological environments. Due to their high specific surface area and strong chemical reactivity, they are prone to oxidative reactions, which can compromise their magnetic properties and dispersibility (Daya et al., 2022). In addition, bare IONPs tend to aggregate and exhibit lipophilicity, making them highly susceptible to binding with lipophilic domains of plasma proteins upon entering the bloodstream. This process, known as opsonization, significantly limits their biomedical applications. Therefore, surface modification with hydrophilic coatings is essential to enhance their colloidal stability and biocompatibility (Solar et al., 2015). Organic polymers such as polyethylene glycol, chitosan (CS), bovine serum albumin (BSA), polydopamine (PDA), and dextran are commonly employed as surface coatings. Compared with inorganic coatings like silica, these organic materials offer superior biocompatibility and biodegradability. They can effectively inhibit undesirable nanoparticle aggregation *in vivo*, thereby maintaining favorable dispersibility and water solubility of IONPs (Shubayev et al., 2009; Jeon et al., 2018).

Despite the promising applications of IONPs in molecular imaging and precision oncology, their toxicological limitations and long-term biosafety remain major obstacles to clinical translation. The primary clearance pathway of IONPs *in vivo* involves the mononuclear phagocyte system, particularly Kupffer cells in the liver and macrophages in the spleen, which may lead to chronic organ burden (Suh et al., 2024). Once internalized into lysosomes, IONPs undergo gradual degradation, releasing iron ions that are subsequently integrated into the systemic iron metabolism through transferrin and ferritin pathways. However, prolonged exposure or high-dose administration may result in local or systemic iron overload, characterized by oxidative stress, lipid peroxidation, and cellular dysfunction (Siddiqui et al., 2023). Moreover, the free iron released during degradation can catalyze Fenton reactions to generate hydroxyl radicals, inducing DNA damage, protein oxidation, and lipid membrane injury. These effects are particularly pronounced in individuals with oxidative stress or metabolic imbalance. In addition, degradation intermediates or unstable forms of IONPs may trigger unexpected biological effects, while the accumulation of non-degradable particles could impair autophagy and lysosomal degradation, disrupting intracellular homeostasis (Jacinto et al., 2025).

IONPs can also elicit immune activation and inflammatory responses. Their biological interactions are strongly influenced by physicochemical properties such as particle size, surface charge, coating chemistry, and protein corona formation. Positively charged or poorly protected nanoparticles tend to induce complement activation-related pseudoallergy, promoting pro-inflammatory cytokine release and recruiting neutrophils and macrophages, which may result in transient fever, hypotension, or even chronic low-grade inflammation (Szebeni, 2024). These immune-mediated effects not only compromise biosafety but also alter biodistribution and therapeutic efficacy. Furthermore, IONPs may induce macrophage polarization toward the M1 phenotype, which, in some cases, could impair tissue repair and regeneration, highlighting the need for precise surface engineering strategies.

Although numerous studies have been conducted, data regarding the long-term toxicity, biodegradation, and clearance mechanisms of IONPs remain limited and inconsistent, with results varying significantly due to differences in synthesis methods and surface coatings. Short-term animal studies generally indicate a wide safety margin at low doses, whereas repeated or high-dose administration has been associated with hepatosplenomegaly, elevated transaminase levels, and mild tissue fibrosis (Haase et al., 2024). Renal clearance is primarily limited to ultra-small particles (<5–10 nm) or dissolved iron species, while larger particles are retained and gradually metabolized in the liver and spleen. Therefore, particle size distribution, dosage, colloidal stability, and surface modification remain key determinants of *in vivo* fate and safety. To minimize adverse effects and facilitate clinical translation, future research should systematically evaluate acute and chronic toxicity across different dosing regimens, with a focus on hepatic and renal function, hematological parameters, and histopathological changes. In parallel, *in vivo* tracking techniques should be employed to quantitatively assess organ distribution, degradation, and clearance kinetics, alongside monitoring immune activation, iron metabolism disorders, and oxidative stress biomarkers. The design of IONPs should prioritize biodegradable, biocompatible, or metabolically transformable materials, incorporating inert or stealth coatings to reduce nonspecific uptake by the mononuclear phagocyte system and inflammatory activation. Furthermore, the establishment of standardized toxicological evaluation systems and long-term follow-up mechanisms will be essential to verify the safety of IONPs and support their eventual clinical implementation.

## 3 Tumor-targeting mechanisms of IONPs

Given the superparamagnetic nature of IONPs, they can be guided and localized using an external magnetic field. Moreover, the unique pathophysiological features of solid tumors such as melanoma—including immature vasculature with defective endothelial linings and basement membranes, as well as impaired lymphatic drainage—further facilitate the targeted accumulation of IONP-based delivery platforms at tumor sites. Simultaneously, they can also be passively and progressively retained within the tumor microenvironment (TME) via the enhanced permeability and retention (EPR) effect (Shi et al., 2020; Zeng et al., 2024). The EPR effect plays a pivotal role in passive targeting and is influenced by multiple factors, including tumor location and type, vascular perfusion, the degree of vascular leakiness, and the physicochemical properties of nanoparticles such as size and morphology (Wu, 2021; Ikeda-Imamuku et al., 2022).

However, due to the variability in melanoma localization and the heterogeneity of local blood perfusion, the magnitude of the EPR effect varies significantly across different TME. This inconsistency limits the specificity of nanoparticle accumulation when relying solely on passive or magnetically guided targeting strategies, thereby restricting the clinical translation of multifunctional nanoclusters for melanoma treatment. To address this limitation, active targeting strategies have been developed, wherein ligands specific to overexpressed receptors on melanoma cells are conjugated to the surface of IONPs to achieve receptor-mediated endocytosis. Currently, the most commonly employed approach for engineering actively targeted IONPs in melanoma diagnostics and therapeutics involves covalent or non-covalent conjugation with monoclonal antibodies (mAbs), folic acid (FA), peptides, nucleic acid aptamers, or small molecule ligands (Kim et al., 2023; Wu, 2022; Zhi et al., 2020). Notably, multivalent nanoprobes can carry multiple binding sites for mAbs, thereby enhancing targeting specificity and, in many cases, preserving the pro-apoptotic activity of the antibodies. Nevertheless, the use of full-length antibodies may elicit undesirable immune responses. To mitigate this risk, functional antibody fragments (e.g., Fab fragments) or synthetic peptides with retained bioactivity have been proposed as alternatives (Opolka-Hoffmann et al., 2021). These strategies not only reduce the risk of immunogenicity but also improve the *in vivo* stability and targeting efficiency of the nanoprobe systems. Despite these advances, the complex interactions between nanomedicines and melanoma pathophysiology remain incompletely understood. The delivery efficiency and therapeutic outcomes of nanomedicines in clinical settings are still uncertain. Therefore, further investigations are required to explore clinically relevant delivery strategies and develop melanoma models that better recapitulate human physiology—such as humanized mouse models—to gain comprehensive insights into the nanoparticle–tumor interaction mechanisms.

## 4 IONPs in melanoma therapy

### 4.1 Targeted drug therapy

Chemotherapy has long served as a cornerstone in the systemic treatment of melanoma, whether as monotherapy or in combination regimens. However, its overall efficacy remains limited (Anand et al., 2023). Dacarbazine (DTIC) is currently the only chemotherapeutic agent approved by both the FDA and the European Medicines Agency for the treatment of advanced malignant melanoma. It has also been widely used as the standard control in numerous clinical trials evaluating novel therapeutics. Nonetheless, DTIC exhibits significant therapeutic limitations. For example, in a clinical study involving 64 patients, the objective response rate for DTIC was only 7%, indicating a very low response rate when used as a single agent (Johnson et al., 2016). Other chemotherapeutic agents, including temozolomide, fotemustine, paclitaxel, and nitrosoureas, have also been evaluated in melanoma treatment. While some patients showed transient responses, the majority of studies have demonstrated that these agents exert minimal impact on overall survival, and their therapeutic effects are often short-lived and difficult to sustain (Luke and Schwartz, 2013). Chemotherapy for melanoma is further hindered by several major challenges, such as systemic toxicity, frequent severe adverse effects, and the widespread occurrence of multidrug resistance (MDR), all of which limit its widespread use in the treatment of advanced melanoma (Li et al., 2018). Nanocarrier systems have emerged as promising alternatives due to their attractive properties, including facile functionalization, enhanced drug targeting, and controlled release capabilities. Among them, polymeric nanocarriers based on IONPs have been the most extensively studied. These platforms offer high drug loading capacity and excellent physicochemical stability. By functionalizing their surfaces with peptides, mAbs, or small-molecule ligands, such nanocarriers can specifically recognize melanoma cells or components of the TME. This not only prolongs the systemic circulation half-life of chemotherapeutic agents and minimizes unintended interactions or off-target toxicity, but also helps to overcome P-glycoprotein (P-gp)-mediated drug resistance, enabling selective accumulation at tumor sites. Furthermore, IONPs can achieve sustained and controlled drug release in response to various external or internal stimuli (e.g., pH, temperature, or tumor-specific enzymes) and targeting mechanisms, thereby increasing the intratumoral concentration of chemotherapeutic agents and reducing damage to healthy tissues (Li et al., 2018; Li et al., 2017).

Over the past few years, various anticancer drugs used for melanoma treatment, such as paclitaxel, dacarbazine, and doxorubicin (DOX), have been combined with IONPs to achieve targeted delivery and enhanced therapeutic efficacy (Sun et al., 2023). Toderascu et al. (2023) developed a novel nanodrug delivery system by coating DOX-loaded iron oxide (Fe_3_O_4_) nanoparticles with L-cysteine (L-Cys), which significantly improved the colloidal stability of IONPs in aqueous media. To systematically evaluate the drug release performance of Fe_3_O_4_-L-Cys-DOX nano-particles, two buffer systems were designed to simulate different biological conditions. Phosphate-buffered saline (PBS, pH 7.4) was used to mimic the normal physiological environment, while citrate buffer (pH 3.0) represented the acidic tumor microenviron-ment. Release profiles demonstrated that within the first 2 h, the release rate of DOX from Fe_3_O_4_-L-Cys-DOX in physiological pH was approximately 10–15 μM/h, whereas under acidic citrate buffer conditions, the release rate doubled compared to physiological conditions. Subsequent *in vitro* cellular assays validated the antitumor activity of this nanoplatform against melanoma cells. It was observed that Fe_3_O_4_-L-Cys-DOX nanoparticles were rapidly internalized by human A375 and murine melanoma cells within 3 h of treatment. After 48 h of incubation, the nanoparticles induced substantial apoptosis, leading to near-complete tumor cell death. This highlights the potential of Fe_3_O_4_-L-Cys-DOX as an efficient drug delivery vector for melanoma therapy. However, this study was limited to *in vitro* testing and did not evaluate the specificity of tumor targeting *in vivo*. Several studies have reported the conjugation of anticancer drugs to IONPs via monoclonal mAbs, ligands, and peptides to achieve selective and precise targeting of melanoma cells *in vivo* (S. V. et al., 2024). Nevertheless, considering melanoma’s superficial location, magnetic drug targeting is regarded as a more efficient alternative strategy. Under the guidance of an external magnetic field, this approach can significantly enhance drug bioavailability at the tumor site while reducing systemic dosage and associated toxicities. In recent years, extensive research has focused on developing and optimizing magnetic drug targeting systems to realize more effective and controllable therapeutic outcomes (Ezike et al., 2023). Cengelli et al. (2009) employed a bifunctional linker to covalently conjugate camptothecin (CPT), a potent topoisomerase I inhibitor, to amino-functionalized polyethylene glycol (PVA)-coated ultrasmall superparamagnetic iron oxide (USPIO) nanoparticles. This design leveraged the bifunctionality of a dicarboxylic acid linker, attaching CPT via a biodegradable ester bond to one end, and anchoring the other end to amino-PVA via an amide bond, thus achieving stable drug loading and cell-specific release. The drug was linked to biocompatible USPIO through an ester bond that is specifically cleaved by intracellular esterases, triggering drug release upon cellular internalization. *In vitro* assays demonstrated that CPT-USPIO exhibited significant cellular uptake and antiproliferative effects in human melanoma cell lines, resulting in marked reductions in metabolic activity and DNA synthesis. CPT-USPIO is primarily localized within intracellular lipid vesicles, indicating uptake predominantly via endocytosis. Furthermore, the application of an external static magnetic field significantly enhanced the cellular uptake of this nanosystem by melanoma cells, suggesting promising potential for *in vivo* magnetic targeting delivery.

Currently, nanoparticle-based drug delivery systems are typically administered via invasive routes such as intravenous injection or intramuscular implantation, often formulated as hydrogels or suspensions. This administration approach necessitates careful consideration of the nanoparticles’ cytotoxicity, biodegradability, and their long-term fate within the body. Indeed, poor *in vivo* stability of nanoparticle systems remains one of the major obstacles limiting their clinical translation. For melanoma treatment, transdermal delivery presents a promising alternative to circumvent these limitations. Studies have shown that certain specialized nanoparticles are capable of penetrating hair follicles and the stratum corneum to reach melanoma cells (Baroli et al., 2007). Rao et al. (2015) covalently linked DOX to the surface of IONPs via pH-sensitive amide bonds, fabricating ultra-small IONPs loaded with the chemotherapeutic agent epirubicin (Epi). These Epi-IONP composites exhibited excellent magnetic properties and demonstrated pH-responsive drug release within a pH range of 4.5–6.8, corresponding to the acidic microenvironment of tumor tissues and intracellular endosomes/lysosomes. The Epi-IONP system was shown to penetrate deep into the skin via the follicular route, bypassing the stratum corneum under the influence of an external magnetic field. *In vitro* cytotoxicity assays revealed good biocompatibility of IONPs with human keratinocyte HaCaT cells, while significantly inhibiting melanoma cell proliferation, suggesting that IONP-based carriers hold potential as transdermal chemotherapeutic candidates for targeted melanoma therapy. Despite these encouraging *in vitro* results, several challenges remain for clinical translation. The long-term biosafety, metabolic pathways, and potential immunological effects of IONPs *in vivo* require thorough investigation. Moreover, maintaining the pH-responsive release behavior of nanoparticles in the complex *in vivo* environment, as well as achieving effective tumor tissue penetration and targeted accumulation, are critical issues yet to be fully addressed. Future efforts should focus on optimizing particle size, surface charge, and ligand modification strategies to further enhance the clinical applicability of IONPs in precise melanoma therapy.

### 4.2 Hyperthermia therapy

In addition to their role as drug carriers for targeted melanoma chemotherapy, IONPs have also been widely explored for magnetic hyperthermia therapy (MHT) and photothermal therapy (PTT). The unique hypoxic and acidic microenvironment within tumor tissues alters the thermal sensitivity of tumor cells, rendering them significantly more susceptible to heat-induced stress than normal cells. This makes tumor cells particularly vulnerable to therapies that employ localized thermal energy to induce cell damage (Bala et al., 2023). The therapeutic benefits of hyperthermia include disruption of DNA repair mechanisms, enhancement of cellular metabolic activity, and stimulation of anti-tumor immune responses through multiple pathways (Chao et al., 2019).

In addition to serving as drug carriers for targeted chemotherapy of melanoma, IONPs have been extensively investigated in MHT, sonothermal therapy (STT), and PTT. The unique hypoxic and acidic TME alters the thermal sensitivity of tumor cells, rendering them significantly more susceptible to heat-induced stress compared with normal cells. This characteristic makes tumor cells particularly vulnerable to therapeutic strategies that induce cellular damage through localized thermal energy (Bala et al., 2023). The therapeutic advantages of hyperthermia include the disruption of DNA repair mechanisms, enhancement of cellular metabolic activity, and activation of antitumor immune responses through multiple pathways (Chao et al., 2019). Moreover, IONP-based cryoablation technology has recently attracted attention for its ability to induce efficient physical destruction of tumor tissue by promoting ice nucleation and growth at low temperatures, accelerating intra- and extracellular ice formation, and thereby offering a promising approach for combined thermo-cryo therapy in melanoma.

#### 4.2.1 Magnetic hyperthermia therapy

MHT is an emerging therapeutic approach that employs functionalized magnetic nanoparticles (MNPs) to selectively accumulate in tumor tissues, where localized heating is induced under AMF via magnetic loss mechanisms. Upon exposure to AMF, MNPs produce heat via Néel and Brownian relaxation mechanisms, leading to an increase in the local microenvironmental temperature by at least 5 °C (Ilg and Kröger, 2020). This thermal effect can result in protein denaturation, disruption of the cell membrane structure, and cytoskeletal damage within tumor cells, thereby inducing apoptosis. Studies have shown that when the local temperature exceeds 42 °C, heat-sensitive tumor cells can be effectively ablated while minimizing damage to surrounding healthy tissues (Repasky et al., 2013). Currently, the heating performance of MNPs under AMF is typically quantified using SAR or specific loss power (Salmanian et al., 2021). From a materials design perspective, the optimization of heating efficiency primarily depends on the regulation of nanoparticle size and morphology. For instance, IONPs have been widely validated for their excellent magnetic-to-thermal energy conversion efficiency (Gomes et al., 2023). The heat generation capacity of MNPs in AMF is influenced not only by their intrinsic properties—such as particle size, crystalline structure, and magnetic responsiveness—but also by extrinsic factors including the frequency and amplitude of AMF, as well as heat dissipation mediated by local blood flow in the targeted tissue (Bellizzi and Bucci, 2010).

Melanoma remains challenging to treat due to its inherent resistance to radiotherapy and multiple chemotherapeutic agents, which severely limits the efficacy of conventional therapies. In recent years, MHT mediated by IONPs has demonstrated promising potential to overcome these limitations. IONPs not only enhance the therapeutic response by increasing radiosensitivity but also enable precise targeting of tumor tissues through their magnetic properties. Blanco-Andujar et al. (2016) employed citric acid-coated IONPs to perform MHT on the human melanoma cell line DX3 and systematically investigated the underlying mechanisms of cell death induced by this approach. Using dual fluorescent labeling with Annexin V-fluorescein isothiocyanate (FITC) and DRAQ7/propidium iodide (PI), the study enabled real-time dynamic imaging and monitoring of apoptosis and necrosis in both suspension and adherent monolayer *in vitro* cell models. The results revealed a delayed onset of AMF-induced cell death, with the rate and extent of cytotoxicity being strongly correlated with the thermal load applied to the cells. Under moderate thermal load, phospholipid translocation across the plasma membrane was activated several hours before cell rupture, without detectable changes in the extracellular temperature, suggesting that cell death was predominantly driven by intracellular heating rather than external thermal exposure. *In situ* imaging of monolayer cells confirmed that the predominant mode of death was apoptosis, as the Annexin V signal consistently appeared before or concurrently with nuclear staining by DRAQ7, and the spatial distribution of dying cells was random, with no evidence of a “bystander effect.” Under low thermal stress, the proportion of FITC-positive cells was significantly higher than that of PI-positive cells, indicating a slow and controllable initiation of cell death. In contrast, under high thermal load, the ratio of apoptotic to necrotic cells approached equilibrium, reflecting an accelerated death process. This study is the first to systematically elucidate the temporal and kinetic characteristics of AMF-induced programmed cell death in melanoma cells, highlighting the potential of MHT as a minimally invasive and clinically translatable therapeutic strategy for melanoma.

However, during MHT, the parameters of the applied AMF must be carefully considered, as they significantly influence the heating efficiency. Numerous studies have demonstrated that hyperthermia induced under conventional sinusoidal AMFs with varying field strengths and frequencies can exert substantial cytotoxic effects on cancer cells (Vilas-Boas et al., 2020). Rego et al. (2020) enhanced MHT efficacy by utilizing amino-silane–coated IONPs with a positive surface charge. By analyzing the heating profiles of the IONPs and calculating their SAR, they identified two AMF parameter sets with suitable absorption ratios for *in vitro* experimentation. Among them, an AMF of 309 kHz and 300 G achieved high heating efficiency with minimal risk. In subsequent *in vivo* experiments, repeated MHT sessions resulted in near-complete tumor regression, and follow-up using multimodal imaging confirmed the absence of tumor recurrence. Souiade et al. (2023) systematically compared the effects of different AMF waveforms on IONP-mediated hyperthermia and highlighted the crucial role of waveform modulation in tumor cell ablation. In a melanoma cell model, they assessed the heating performance and cytotoxicity of IONPs under three AMF waveforms: sinusoidal, trapezoidal, and near-square wave. The results showed that IONPs excited by near-square wave AMF exhibited significantly enhanced thermal output and were more effective in inducing melanoma cell death compared to those under traditional sinusoidal or trapezoidal waveforms. These findings suggest that, beyond the conventional optimization of IONPs size, morphology, and surface characteristics, engineering the waveform of the AMF itself plays a vital role in improving MHT efficacy. This approach represents a promising direction for future research in cancer nanotherapy.

Certainly, thermal effects are not the sole mechanism by which MHT induces tumor cell death. Under AMF, IONPs can also trigger a range of non-thermal pathways, such as increasing lysosomal membrane permeability (Domenech et al., 2013; Connord et al., 2015). This process is closely associated with elevated intracellular levels of reactive oxygen species (ROS) and enhanced cytoplasmic activity of cathepsin D, a lysosomal protease. Lysosomes, as organelles rich in hydrolytic enzymes, rely on the integrity of their membranes to maintain cellular homeostasis. Once the lysosomal membrane is compromised, the release of lysosomal enzymes into the cytoplasm can activate downstream signaling cascades, ultimately leading to lysosome-dependent cell death (Serrano-Puebla and Boya, 2018). Following MHT treatment, a significant decrease in cell viability has been observed, accompanied by upregulation of pro-caspase expression, increased ROS production, and altered mRNA levels of proliferation-associated markers such as Ki-67. These findings suggest that MHT-induced cytotoxicity involves multifactorial synergistic mechanisms. On one hand, the AMF promotes the conversion of electromagnetic energy into heat by IONPs. On the other hand, the oscillation of IONPs within cellular organelles generates mechanical forces that disrupt plasma and lysosomal membrane structures, thereby initiating death signaling cascades (Souiade et al., 2023). Therefore, current perspectives suggest that the therapeutic efficacy of MHT is not solely dependent on localized hyperthermia but also stems from mechanical disruption of cellular and organelle membrane integrity. This non-thermal mechanism provides a more complex and effective biological foundation for the anti-melanoma potential of MHT (Lopez et al., 2022). Nevertheless, MHT still faces several challenges, including the limited heating efficiency of nanomaterials, suboptimal biocompatibility, uneven heat distribution, equipment-related constraints, and an incomplete understanding of its underlying mechanisms. Future research should focus on the development of high-performance nanomaterials, precise thermal control strategies, optimized combination therapies, and advanced device engineering to facilitate clinical translation and personalized applications.

#### 4.2.2 Photothermal therapy

PTT is a non-invasive therapeutic modality that employs photothermal agents (PTAs) to convert light energy—typically from external sources such as near-infrared (NIR) light—into thermal energy to induce tumor cell ablation. Studies have demonstrated that IONPs can absorb NIR light, promoting the excitation of electrons from the ground state to an excited state, and subsequently release the energy in the form of heat through non-radiative decay. Owing to their favorable biostability and tumor-targeting properties, IONPs serve as excellent PTAs, exhibiting strong NIR absorption and high photothermal conversion efficiency (Busquets et al., 2020). Beigi et al. (2022) investigated the *in vivo* photothermal ablation performance of PDA-coated IONPs in a murine melanoma model. A Q-switched ruby laser with a wavelength of 694 nm was employed for irradiation. The results showed that after four treatment sessions, tumor volume in the treatment group was reduced by 74% compared with the control group, and no significant adverse effects were observed. Both the concentration of nanoparticles and the laser power density significantly influenced the temperature rise; as the concentration of nanoparticles increased, the efficiency of light energy absorption improved accordingly, thereby enhancing the photothermal ablation effect. Pandesh et al. (2021) synthesized core–shell structured magnetic gold nanoparticles (Fe_3_O_4_@Au) and applied them for magnetically targeted PTT in melanoma. The average hydrodynamic diameter of the Fe_3_O_4_@Au nanoparticles was 37.8 nm. In the *in vivo* experiments, five groups were established: a control group, a laser-only group, a nanoparticles (NPs)-only group, an NPs + laser group, and an NPs + magnetic targeting + laser group. Over 2 weeks, the average tumor volume fold increases were 47.3, 45.3, 32.8, 19.9, and 7.7, respectively. No significant changes in the average body weight of mice were observed across all groups, indicating that the combination of magnetic field-assisted nanoparticle targeting and laser irradiation exerted a pronounced inhibitory effect on melanoma proliferation.

Melanoma is characterized by its highly invasive nature, readily breaching the basement membrane and spreading into the deep dermis, subcutaneous tissue, and even lymphatic and hematogenous systems. This biological characteristic poses significant challenges for PTT, primarily due to the relatively limited penetration depth of lasers in the first near-infrared window (NIR-I; 700–900 nm), resulting in insufficient ablation of malignant tumors outside the irradiation zone. Wu et al. (2020) proposed the use of the second near-infrared window (NIR-II; 1000–1700 nm), particularly the NIR-IIa sub-window (NIR-IIa; 1300–1400 nm), which exhibits greater tissue penetration depth and demonstrates superior antitumor efficacy in deep-seated tissues *in vivo*. This suggests that combining NIR-II window lasers with IONPs enables real-time monitoring and targeted therapy of deep tumor tissues. However, light penetration remains insufficient for deeply located tumors. To overcome the inherent penetration limitations of conventional PTT, advanced approaches such as enlarged laser spot PTT, optical clearing techniques for skin tissue, fiber-optic–assisted PTT, and associated detection devices are currently under development. Additionally, the design of “activatable” IONPs with tumor microenvironment-responsive photothermal properties represents a promising strategy to enhance PTT efficacy. Nevertheless, IONPs face numerous biological barriers; for instance, circulating IONPs within the vascular system are prone to recognition, uptake, and degradation by the mononuclear phagocyte system, which reduces their accumulation and localization at tumor sites, thereby limiting their application as effective PTAs. Zhang et al. (2018) developed macrophage membrane-coated magnetic nanoparticle clusters for efficient IONPs delivery and antitumor therapy. By cloaking nanoparticles with macrophage membranes, these constructs effectively evade phagocytosis by the RES *in vivo*, while exploiting the inherent tumor-homing and adhesion properties of macrophages to achieve targeted enrichment within the melanoma microenvironment. This strategy significantly prolongs systemic circulation time and enhances targeting capability. However, key challenges remain for clinical translation, including whether membrane-coated nanoparticles might induce oncogene expression and how to maintain membrane stability and antimicrobial properties during fabrication. Addressing these issues is critical for the safe and effective application of biomimetic nanocarriers in cancer therapy.

#### 4.2.3 Sonothermal and cryothermal therapies

IONPs have shown remarkable potential as enhancers in focused ultrasound-mediated STT for melanoma treatment. By improving local acoustic energy deposition, IONPs enable multimodal, image-guided, and precisely controlled interventions. Studies have demonstrated that uniformly dispersing IONPs within tissue-mimicking media or delivering them in the form of particle-stabilized Pickering emulsions can enhance ultrasonic attenuation and scattering in tumor regions, thereby achieving a significantly higher localized temperature rise without increasing the overall acoustic power. In particular, Pickering emulsions form highly absorptive interfacial regions that generate more intense and spatially confined heating gradients (Ratajczak et al., 2025). The STT enhancement mechanism of IONPs involves several key aspects. First, interactions between nanoparticles and the surrounding medium increase viscous and thermal losses, thereby enhancing acoustic scattering and absorption at particle interfaces. Second, IONPs can serve as effective cavitation nuclei, facilitating the initiation of inertial or stable cavitation. When exposed to an acoustic field, cavitation bubbles undergo nucleation, growth, and collapse, producing transient hotspots characterized by ultrahigh temperatures (up to several thousand degrees Celsius) and pressures (hundreds of atmospheres), which markedly improve the efficiency of acoustic-to-thermal conversion (Li et al., 2022). Additionally, owing to their magnetic responsiveness, IONPs can generate magnetothermal effects under an alternating magnetic field, creating synergistic heating with ultrasound and enabling spatiotemporally precise energy control. For melanoma therapy, IONPs possess a unique magnetic targeting advantage—external magnetic field gradients can effectively accumulate and immobilize the particles at cutaneous or subcutaneous tumor sites, improving local treatment efficacy while minimizing off-target damage. Combined with MRI or ultrasonic thermometry, real-time visualization of particle distribution and temperature evolution can be achieved, ensuring enhanced safety and dose controllability. More importantly, compared with PTT, STT exhibits superior tissue penetration, tighter energy focusing, and minimal interference from skin pigmentation, thereby allowing noninvasive and precise ablation of deep-seated or metastatic melanoma. Collectively, these attributes make IONP-mediated STT a highly promising strategy for precision melanoma treatment. In contrast to heat-based therapies, IONP-mediated cryoablation relies on “cold-induced injury” as its primary mechanism, achieving tumor destruction through physical disruption of tissue architecture (Ye et al., 2017). This approach induces local tumor necrosis by rapid cooling, which triggers intracellular ice crystal formation, mechanical rupture of cell membranes, and microvascular occlusion. Nanomaterials have been shown to function as heterogeneous ice nucleation agents during freezing, accelerating ice formation and promoting intracellular crystal growth and propagation, thereby significantly enhancing the cryogenic effect. For instance, surface-functionalized nanostructures coated with polydopamine or CS can improve hydrophilicity and interfacial activity, thereby increasing nucleation rates and amplifying cell damage (Hou et al., 2021). Moreover, integrating phase-change materials or high-thermal-conductivity metallic nanostructures with photothermal nanoparticles enables synergistic energy regulation and controlled drug release during freeze–thaw cycles, leading to enhanced comprehensive ablation of melanoma (Hou et al., 2020). Compared with thermally driven nanosystems, cryoablation-associated nanomaterials exhibit distinct physical characteristics, including the ability to promote rapid and ordered ice nucleation, modulate ice crystal morphology, induce membrane disruption through phase interference, and achieve controlled drug release while minimizing collateral freezing injury to adjacent tissues. Typical nanomaterials used in cryoablation include inorganic particles such as silver, magnesium oxide, and iron oxide. Among them, IONPs—owing to their excellent magnetic responsiveness and nucleation activity—can effectively regulate ice crystallization kinetics, synchronize intra- and extracellular freezing, and thereby significantly increase tissue freezing depth and cell lysis efficiency (Kwak et al., 2022). These properties offer new research directions for efficient cryoablation and combination therapies in melanoma. However, given the highly invasive and treatment-resistant nature of melanoma, investigations into IONP-based sonothermal and cryoablation technologies remain limited, and clinical validation is even scarcer. Future studies should focus on systematic preclinical and clinical evaluations to further assess the safety, controllability, and overall therapeutic efficacy of these approaches in melanoma management.

### 4.3 Photodynamic and chemodynamic therapies

Photodynamic therapy (PDT), as an alternative therapeutic modality for melanoma, offers advantages such as minimal invasiveness, the capability to eradicate occult cancerous lesions, and relatively mild toxicity. PDT employs photosensitizers (PSs) that can be activated by lasers of specific wavelengths as substrates, generating highly oxidative ROS to kill melanoma cells. This process triggers oxidative stress responses in cellular signaling pathways or modulates gene expression within the TME, leading to phototoxic effects (Montaseri et al., 2021). PDT primarily involves two oxygen-dependent mechanisms: Type I and Type II photochemical reactions. In the Type I pathway, the excited triplet state PSs transfers energy to surrounding biomolecules, reacting with substrates in the tumor tissue via hydrogen atom or electron transfer to produce free radicals (Kwiatkowski et al., 2018). These radicals subsequently react with molecular oxygen to form highly oxidative ROS, including superoxide anions and hydroxyl radicals (⋅OH), which ultimately activate multiple cell death pathways such as apoptosis, necrosis, and autophagy (Nkune et al., 2021). In contrast, the Type II mechanism involves direct energy transfer from the excited PSs to molecular oxygen, generating singlet oxygen (^1^O_2_), a highly reactive form of ROS (Nkune et al., 2021). Regardless of the pathway, the ROS and ^1^O_2_ produced induce oxidative damage to key intracellular biomolecules, including protein denaturation, lipid peroxidation, and structural disruption of organelles such as mitochondria and the endoplasmic reticulum, thereby triggering diverse modes of cell death (Amos-Tautua et al., 2019). However, the clinical translation of PDT has been hindered by several factors, including the significant toxicity and low ROS generation efficiency associated with first- and second-generation PSs, as well as poor solubility and unfavorable pharmacokinetic profiles. Moreover, systemic administration of PSs often results in insufficient selective accumulation within tumor tissues, leading to a spectrum of non-specific side effects during light irradiation. For instance, during PDT for melanoma, current PSs lack high specificity toward melanoma cells, and the irradiation typically covers a large area, including surrounding normal tissues, thereby increasing the risk of damage to healthy lung tissue and other organs (Grossman et al., 2011). Therefore, the development of innovative strategies to enhance the targeted delivery efficiency of PSs to melanoma cells, thereby significantly improving the therapeutic efficacy of PDT, remains a major focus and challenge for researchers in this field.

Clinically, third-generation PSs are being developed by conjugating second-generation PSs with nanoparticles that possess favorable biocompatibility and tumor-targeting properties, thereby enhancing ROS generation efficiency, hydrophilicity, pharmacokinetics, and desired *in vivo* biodistribution (Hu et al., 2021; Przygoda et al., 2023). Various nanoparticle platforms have been engineered for systemic PS delivery, including micelles, dendrimers, liposomes, polymeric nanoparticles, and silica nanoparticles. Among these carriers, IONPs not only effectively deliver hydrophobic PSs but also enable magnetic resonance imaging (MRI)-guided targeted theranostics, providing unique advantages for the synergistic application of PDT and imaging. Nistorescu et al. (2021) synthesized γ-Fe_2_O_3_ nanoparticles via laser pyrolysis and functionalized their surface with water-soluble tetraphenylporphine sulfonate (TPPS). The ^1^O_2_ quantum yield of the γ-Fe_2_O_3_–TPPS complex was measured to be approximately 60% by phosphorescence detection at 1,270 nm. Upon treating melanoma cells with 0.5–0.75 μg/mL of the γ-Fe_2_O_3_–TPPS complex for 24 h, followed by 1-min blue light irradiation at 405 nm with an intensity of 1 mW/cm^2^, a significant enhancement in ROS generation was observed even at ultra-low light doses. This treatment inhibited cell proliferation and adhesion, induced caspase-3/Bax-mediated apoptosis, and notably downregulated the expression of proliferation-related protein MCM-2 and signaling protein β-catenin, demonstrating a marked PDT efficacy in melanoma cells.

On this basis, chemodynamic therapy (CDT), as a treatment modality relying on endogenous chemical reactions and independent of external light activation, provides an important complementary and synergistic pathway to PDT. CDT utilizes transition metal ions (such as Fe^2+^) to trigger Fenton or Fenton-like reactions in the presence of hydrogen peroxide (H_2_O_2_) within tumor cells, generating highly reactive ⋅OH and other ROS, which in turn induce oxidative stress–mediated apoptosis or necrosis. Given that melanoma tissues often exhibit elevated levels of endogenous H_2_O_2_ and a mildly acidic microenvironment, these features create favorable conditions for the *in situ* catalytic reactions of CDT. The sustained release of Fe^2+^ ions from IONPs within the tumor can continuously promote the conversion of H_2_O_2_ into ⋅OH, enabling a “self-supplied substrate” mode of ROS generation. Mechanistically, PDT and CDT are inherently complementary: while PDT depends on molecular oxygen to generate ^1^O_2_, CDT can convert H_2_O_2_ into both oxygen and ⋅OH through Fenton chemistry, thereby partially alleviating the hypoxia-induced limitation of PDT (Fu et al., 2023). Conversely, the local hyperthermia and photo-oxidation process induced by PDT can facilitate Fe^2+^ release and accelerate CDT reaction kinetics, enhancing ROS generation. Consequently, IONP-based PDT–CDT synergistic systems have emerged as a research hotspot in melanoma nanotherapy. However, ROS-dependent therapeutic strategies remain limited by the TME. The widespread hypoxia in tumors restricts the efficiency of oxygen-dependent processes, especially the Type II PDT reactions (Chen Y. et al., 2023). Moreover, the high intracellular concentration of glutathione (GSH) in tumor cells can effectively scavenge ⋅OH and other ROS, thereby diminishing the cytotoxic efficacy of CDT. These factors collectively make single-mode ROS amplification strategies insufficient for achieving satisfactory clinical outcomes.

To overcome these challenges, recent studies have proposed multiple “ROS amplification and TME modulation” strategies and integrated them into IONP-based nanoplatforms to realize dual or multimodal therapeutic synergy. Deng et al. (2021) introduced oxygen-generating components or vitamin C into IONPs composite systems to relieve tumor hypoxia and continuously supply substrates for the Fenton reaction, thereby markedly enhancing ⋅OH and ^1^O_2_ production and improving CDT-mediated oxidative stress and antitumor efficacy. Similarly, Chen et al. (2019) developed a Fe_3_O_4_-HSA@Lapa nanoplatform with both Fenton-like catalytic activity and GSH-depleting capability, achieving a significant amplification of oxidative stress within tumors. In this system, β-lapachone (Lapa) selectively generates H_2_O_2_ under catalysis by NAD(P)H:quinone oxidoreductase 1, providing substrates for Fenton reactions with Fe^2+^ released from Fe_3_O_4_, leading to efficient ⋅OH production and oxidative damage in the TME. Meanwhile, the release of Lapa further depletes intracellular GSH, weakening antioxidant defenses and reinforcing CDT cytotoxicity.

In summary, the deep integration of PDT and CDT, together with their combination with magnetic and sonothermal therapies, represents one of the most promising directions in IONP-based melanoma treatment research. The key lies in precise modulation of the TME and spatiotemporal control of ROS kinetics. Future studies should focus on reaction kinetics modeling, pharmacokinetic optimization, and clinical safety validation to promote translational application.

### 4.4 Immunotherapy

**FIGURE 2:**
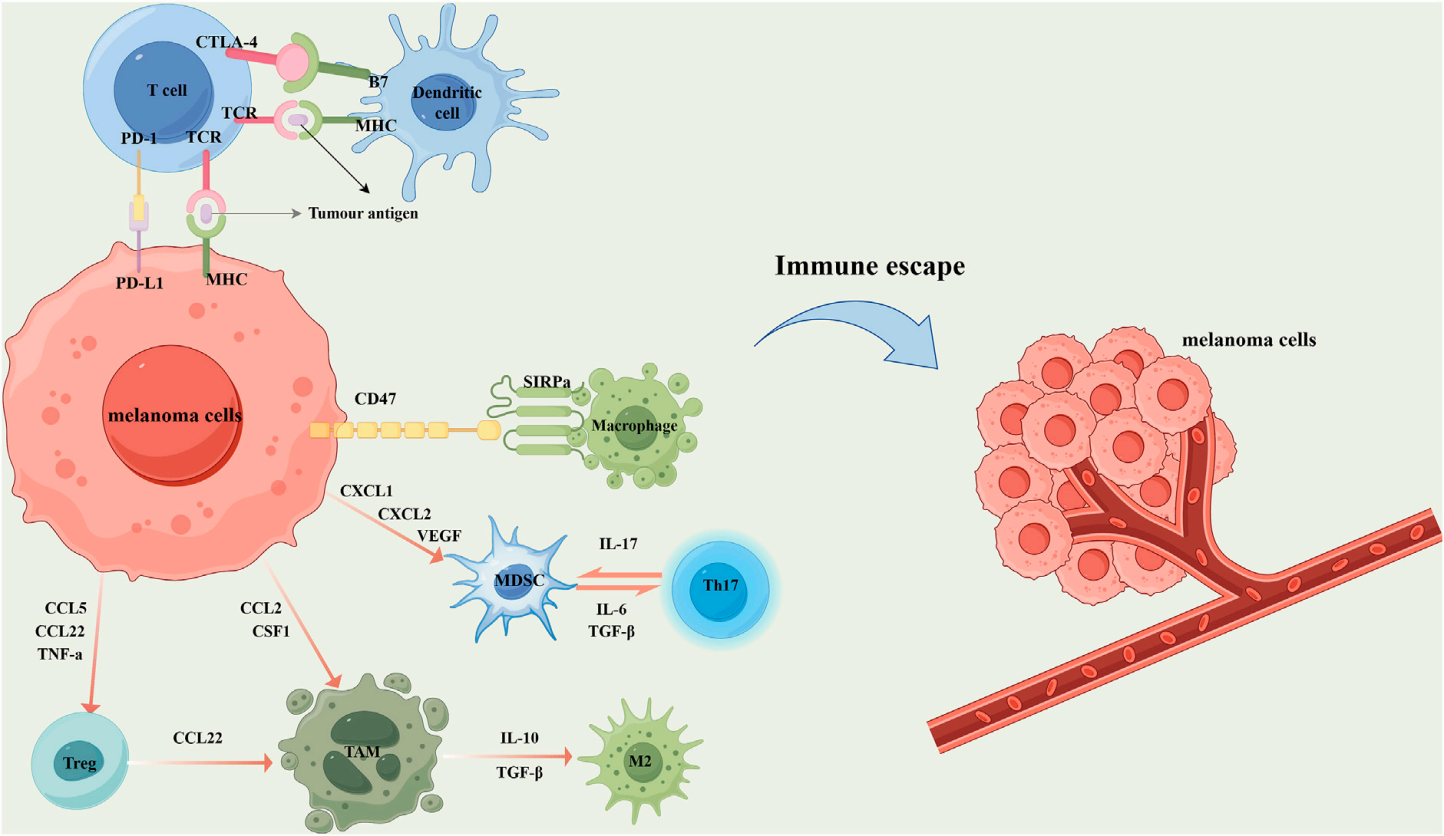
In the local microenvironment of melanoma, tumor cells secrete a variety of chemokines and cytokines to recruit immunosuppressive cells, thereby inhibiting the anti-tumor immune response. Specifically, the overexpression of CD47 on melanoma cells interacts with signal regulatory protein alpha (SIRPα) on the surface of macrophages, transmitting a “don't eat me” signal that suppresses macrophage-mediated phagocytosis. Concurrently, melanoma cells secrete CXCL1, CXCL2, and vascular endothelial growth factor (VEGF) to recruit myeloid-derived suppressor cells (MDSCs). MDSCs further contribute to immune suppression by cooperating with T helper 17 (Th17) cells through the secretion of IL-17, IL-6, and transforming growth factor-β (TGF-β). Melanoma cells also secrete CCL5, CCL22, and TNF-α to attract regulatory T cells (Tregs), and release CCL2 and colony-stimulating factor 1 (CSF1) to recruit tumor-associated macrophages (TAMs). Tregs further promote the accumulation of TAMs by secreting CCL22. Under the influence of IL-10 and TGF-β, TAMs are polarized into the tumor-promoting M2 phenotype, which significantly facilitates melanoma progression, metastasis, and immune tolerance. In addition, melanoma cells express and secrete various immune-regulatory molecules that interact with immune cells, thereby suppressing their cytotoxic activity and promoting immune escape. For example, the high expression of programmed death-ligand 1 (PD-L1) on tumor cells binds to programmed cell death protein 1 (PD-1) on T cells, delivering inhibitory signals that attenuate antigen presentation and T cell activation. Meanwhile, cytotoxic T lymphocyte-associated protein 4 (CTLA-4) on T cells competitively binds to B7 molecules on dendritic cells with higher affinity, blocking the co-stimulatory signals required for T cell activation and proliferation, and thus further dampening anti-tumor immunity. These mechanisms collectively contribute to the formation of an immunosuppressive tumor microenvironment and ultimately lead to melanoma immune evasion.

Melanoma has long been regarded as a highly immunogenic malignancy due to its capacity to elicit robust anti-tumor immune responses (Figure 2). In recent years, immunotherapeutic strategies targeting melanoma immune evasion mechanisms have achieved preliminary clinical success (Shan and Liu, 2025). Immunotherapy facilitates the reduction of tumor size and the extent of local advanced melanoma, thereby converting previously unresectable tumors into surgically operable states. These approaches include cytokine therapy, cancer vaccines, and the adoptive transfer of transient T cells (Long et al., 2023). The core principle lies in activating the host immune system to recognize and effectively eliminate tumor cells, involving a coordinated interplay between innate and adaptive immune responses. Macrophages, as key components of the innate immune system, serve as the first line of defense by clearing pathogens and aberrant cells. They mediate the clearance of senescent, apoptotic, or diseased cells through recognition and phagocytosis, subsequently initiating inflammatory responses and activating downstream effector cells to eradicate pathogens and tumor cells. However, tumor cells overexpress Cluster of Differentiation 47 (CD47) to evade macrophage-mediated phagocytosis. CD47 binds to Signal Regulatory Protein Alpha (SIRPα) on macrophage surfaces, delivering a “don’t eat me” signal that inhibits phagocytic activity (Roelands et al., 2023). CD47 is highly expressed across multiple tumor types, including melanoma, contributing to immune evasion (Tang et al., 2023). Moreover, the TME of melanoma is predominantly infiltrated by tumor-associated macrophages (TAMs) of the pro-tumorigenic M2 phenotype, which further promotes melanoma progression, metastasis, and immune tolerance (Shapouri-Moghaddam et al., 2018). Consequently, immunotherapeutic interventions targeting the CD47–SIRPα axis have garnered increasing attention. Studies have demonstrated that anti-CD47 antibodies can block this immune evasion pathway, restoring macrophage-mediated phagocytosis of melanoma cells (Li B. et al., 2024). Concurrently, inducing the repolarization of TAMs toward the M1 phenotype holds promise for remodeling the TME and enhancing anti-tumor immunity. Existing evidence indicates that IONPs can effectively mediate TAM polarization toward the M1 phenotype (Li M. et al., 2024).

Ferumoxytol, an iron oxide nanoparticle approved by the FDA for the treatment of iron deficiency anemia, has demonstrated significant immunomodulatory potential *in vitro*. It markedly upregulates M1-type TAM markers such as CD86 and tumor necrosis factor-alpha (TNF-α), while suppressing M2-type TAM-associated markers including CD206 and interleukin-10 (IL-10) (Yang et al., 2025). Upon efficient uptake by TAMs, ferumoxytol induces intracellular Fenton reactions that generate abundant ROS, thereby promoting the phenotypic shift from the immunosuppressive M2 subtype to the pro-inflammatory M1 subtype. This ROS-driven phenotypic reprogramming not only enhances the macrophages’ anti-tumor activity but also induces apoptosis in subcutaneous melanoma cells (Nascimento et al., 2021).

However, since CD47 is also widely expressed on normal cells, systemic blockade of CD47 often leads to adverse effects such as anemia. Therefore, strategies targeting CD47 urgently require enhanced tumor specificity to minimize “off-target” damage. Recent studies have developed magnetically guided nanosystems by covalently conjugating anti-CD47 antibodies onto the surface of silica-coated IONPs, enabling their enrichment at metastatic tumor sites and effectively blocking the interaction between tumor cell-expressed CD47 and macrophage SIRPα. This approach significantly enhances macrophage-mediated phagocytosis (Rao et al., 2020). Furthermore, some anti-CD47 antibodies can activate antibody-dependent cellular phagocytosis via their fragment crystallizable (Fc) region, thereby potentiating their anti-tumor efficacy (Osorio et al., 2023).

In addition to modulating TAMs, activation of cytotoxic T lymphocyte (CTL) is also a critical component for enhancing anti-tumor immune responses (Song et al., 2021). “Immunologically cold” tumors, such as melanoma, often exhibit low levels of CTL infiltration and harbor an immunosuppressive TME enriched with various suppressive cell populations, including regulatory T cells (Tregs), myeloid-derived suppressor cells (MDSCs), and M2-polarized TAMs (Wang J. et al., 2024). Moreover, the high expression of immune checkpoint molecules—such as programmed cell death protein 1 (PD-1), its ligand PD-L1, and cytotoxic T lymphocyte-associated antigen 4 (CTLA-4)—further induces CTL exhaustion, limiting their tumor-clearing capacity (Long et al., 2025). DCs, as pivotal bridges between innate and adaptive immunity, possess exceptional capabilities in antigen uptake, processing, and cross-presentation, thereby effectively inducing activation and expansion of CD8^+^ T cells to elicit CTL-mediated anti-tumor effects (My et al., 2024). However, recruitment, maturation, and activation of DCs within the TME are also profoundly suppressed. The formation of co-inhibitory signals via interactions of PD-L1/PD-L2 and CD80/CD86 with PD-1 or CTLA-4 on T cells constitutes a major mechanism underlying immune response attenuation (Heras-Murillo et al., 2024).

In recent years, immune checkpoint inhibitors, such as anti-PD-1, anti-PD-L1, and anti-CTLA-4 antibodies, have been demonstrated to release T cells from their exhausted state and restore cytotoxic functions, significantly improving therapeutic responses in a subset of melanoma patients (Wang K. et al., 2024). Concurrently, the precise delivery of tumor antigens and Toll-like receptor (TLR) agonists to DCs via nanocarrier systems to enhance DCs maturation and antigen-presenting capability has emerged as a promising strategy in next-generation melanoma vaccine development (Zhao et al., 2025). For instance, several studies reported that Fe_3_O_4_ NPs covalently conjugated with tumor antigens can increase DCs uptake and induce their differentiation toward CD103^+^ or CD8α^+^ cross-presenting phenotypes, markedly enhancing CD8^+^ T cell activation efficiency (Shadbad et al., 2021). Luo et al. (2019) designed and synthesized a nanoscale immunocomposite (Fe_3_O_4_-OVA) by covalently coupling ultrasmall Fe_3_O_4_ NPs with the model antigen ovalbumin (OVA), proposing and validating a novel role for Fe_3_O_4_ as a “nano-immunoadjuvant.” This approach integrates antigen stabilization and delivery, DCs activation, and macrophage reprogramming, representing a transition from conventional imaging contrast agents to multifunctional immune nanoplatforms with autonomous immunomodulatory functions. The covalent linkage between Fe_3_O_4_ and OVA significantly improves antigen stability *in vivo*, ensuring effective uptake by antigen-presenting cell (APC). *In vitro*, Fe_3_O_4_-OVA notably upregulated the expression of DCs maturation markers CD40 and CD86 on bone marrow-derived dendritic cells and enhanced T cell production of high levels of interferon-gamma (IFN-γ), thereby eliciting robust cytotoxic T lymphocyte-mediated antitumor immunity. Additionally, Fe_3_O_4_ NPs, through their iron content, participate in Fenton reactions to generate ROS within the tumor immune microenvironment, promoting polarization of TAMs from the immunosuppressive M2 phenotype to the proinflammatory M1 phenotype. This “*in situ* immune remodeling” effect further amplifies the strength and durability of vaccine-induced immune responses. *In vivo* experiments using B16-OVA melanoma-bearing mice demonstrated that Fe_3_O_4_-OVA significantly inhibited tumor growth and prolonged survival. Moreover, vaccination substantially reduced pulmonary metastatic nodules in a melanoma lung metastasis prevention model, indicating its capability not only for tumor therapy but also for inducing durable immune memory and metastasis prevention. Importantly, the nanovaccine exhibited excellent biocompatibility and safety, with no significant histopathological damage or functional abnormalities detected in major organs such as the liver, kidney, and spleen at therapeutic doses. Chiang et al. (2018) constructed an immunomodulatory platform based on IONPs, conjugating immune checkpoint inhibitor anti-PD-L1 antibodies and T cell activation molecules (e.g., anti-CD3 and anti-CD28 antibodies) onto fucoidan–glucan-modified IONPs via acid-sensitive linkers. Guided by an external magnetic field to enhance tumor-site accumulation, this strategy significantly augmented PD-L1 blockade and promoted CTL activation in melanoma-bearing mice, extending median survival from 32 to 63 days. Furthermore, owing to the substantially reduced antibody dosage compared to free antibody treatment, systemic toxicity was markedly decreased. Magnetic nanoparticles thus exhibit multiple advantages in melanoma immunotherapy: serving as immunomodulators to induce TAM repolarization toward M1 phenotype, acting as delivery platforms for antibodies or agonists, and enabling precise tumor or immune cell targeting via magnetic navigation, collectively enhancing immune responses while minimizing systemic side effects.

In addition, MHT, PTT, and cryoablation therapy (CTT) not only rely on physical energy to achieve direct ablation of local tumor cells but also induce immunogenic cell death (ICD), thereby activating systemic antitumor immune responses (Liu et al., 2024). Their common mechanisms include the release of damage-associated molecular patterns (DAMPs), promotion of APC maturation, activation of T-cell effector responses, and establishment of long-term memory immunity.

In MHT, IONPs generate localized thermal stress under an alternating magnetic field, triggering mitochondrial damage and endoplasmic reticulum stress, which leads to the release of ATP, HSP70, and calreticulin as DAMPs. This process promotes DC maturation and M1-type macrophage polarization, thereby activating cytotoxic CD8^+^ T cells and reversing immune suppression (Jiang et al., 2022). Moreover, MHT can upregulate tumor PD-L1 expression, and when combined with immune checkpoint inhibitors, it enhances the antitumor immune response.

PTT induces apoptosis through localized hyperthermia generated by NIR irradiation, while also producing heat shock proteins and inflammatory cytokines that enhance antigen presentation and T-cell recruitment. The combination of PTT with immune adjuvants or matrix-degrading enzymes can further promote DC maturation, improve CTL infiltration, overcome the dense extracellular matrix barrier of solid tumors, and enhance immune cell infiltration and cytotoxicity in the tumor core (Yu et al., 2024).

CTT induces the release of abundant DAMPs through rapid freeze–thaw thermal stress cycles, promoting the differentiation of MDSCs into DCs, enhancing M1 macrophage polarization and Th1-type immune responses. Studies have shown that CTT can regulate the coordinated activation of CD4^+^ and CD8^+^ T cells via the LFA-1/ICAM-1–Notch1 signaling axis, forming an IL-2 positive feedback loop that establishes long-term memory immunity and effectively prevents tumor recurrence and metastasis (Wang S. et al., 2024). Furthermore, combining CTT with adoptive cell therapy can markedly remodel the TME, enhance T-cell clonal expansion and effector functions, and produce a superior systemic antitumor immune response compared with monotherapies.

### 4.5 Combination therapy

Given the highly heterogeneous nature of tumors, single-modality therapeutic strategies often fail to achieve satisfactory efficacy, with limited clinical response rates. To enhance treatment efficiency and overcome resistance mechanisms, current research trends are increasingly focusing on multimodal therapeutic strategies that integrate multiple treatment modalities. These approaches aim to achieve synergistic therapeutic effects and address the limitations associated with conventional monotherapies. Combination therapies appear to be more effective in promoting melanoma ablation than any single treatment modality alone (Yi et al., 2024).

The combination of multiple therapeutic modalities—such as MHT, PDT, radiotherapy, and chemotherapy—has been shown to enhance antitumor efficacy while minimizing adverse effects. Tamura et al. (2022) proposed a novel chemo-thermo-immunotherapy (CTI) strategy that integrates N-propionyl-4-S-cysteaminylphenol (NPrCAP), a melanin biosynthesis substrate, with IONPs. Under the catalytic action of tyrosinase, NPrCAP generates highly reactive radicals, enabling selective cytotoxicity against melanoma cells. Simultaneously, IONPs generate localized hyperthermia under AMF, which not only induces apoptosis but also upregulates heat shock protein expression, thereby enhancing antitumor immune responses. In a murine xenograft melanoma model, this combined treatment significantly inhibited tumor growth at the local site and, notably, elicited immune responses at distant, untreated tumor lesions, demonstrating an “*in situ* vaccination” effect. Further mechanistic studies revealed that CTI therapy facilitated CD8^+^ T cell infiltration into the TME and upregulated the expression of major histocompatibility complex class I (MHC-I) molecules. These findings suggest that CTI therapy has the potential to enhance tumor immunogenicity and convert immunologically “cold tumors” into “hot tumors,” thereby improving the responsiveness of melanoma to immunotherapy. Duval et al. (2019) investigated the mechanisms by which low-dose hyperthermia or moderate-dose radiotherapy modulate immune responses and cytotoxic effects in melanoma cells at both the genetic and proteomic levels. Using a murine melanoma cell model, the authors applied IONP-mediated hyperthermia either alone or in combination with 8 Gy irradiation and systematically evaluated alterations in key immune-related pathways and cell death signals. The results demonstrated that hyperthermia significantly upregulated the expression of immunostimulatory molecules such as HSP70, CXCL10, and CXCL11, as well as innate immune receptors including TLR3 and TLR4. Moreover, suppression of the p38/MAPK axis suggested a reduction in the immune evasion capacity of melanoma cells. The increased expression of CD86 further underscored the induction of ICD by hyperthermia, laying a molecular foundation for subsequent T cell-mediated immune responses. When hyperthermia was combined with radiotherapy, a synergistic increase in the expression of critical immune and apoptotic genes—such as CXCL11, CD86, Fas, and PUMA—was observed. Apoptotic signaling was notably enhanced, with peak activation in the ERK2, CASP3, and MAPK11 pathways, indicating robust engagement of programmed cell death mechanisms. Interestingly, the activation of the CXCL10/CXCL11–CXCR3 axis and TLR3/TLR4 signaling pathways suggested that the combination of hyperthermia and radiotherapy could effectively reshape the tumor immune microenvironment and enhance tumor cell sensitivity to cytotoxic attacks. This hyperthermia-mediated “immune adjuvant” effect provides a strong mechanistic basis for the immunosensitizing role of radiotherapy and highlights the potential of hyperthermia in precisely modulating tumor immunogenicity to improve therapeutic outcomes.

In addition, several studies have explored the combination of PTT with chemotherapy to induce synergistic therapeutic effects. This combinatorial strategy not only accelerates cellular biochemical reaction rates via thermal effects, thereby promoting local blood circulation in melanoma tissues and enhancing the accumulation and penetration efficiency of chemotherapeutic agents, but also increases cell membrane permeability to facilitate drug uptake. Furthermore, it can overcome chemotherapy resistance in melanoma cells by inhibiting the activity of drug efflux-related transport proteins (Krawczyk et al., 2011). Elevated temperatures can also augment the cytotoxic effects of chemotherapeutic agents through mechanisms such as increased ROS production and inhibition of DNA repair pathways (Yoncheva et al., 2019). Therefore, compared to monotherapies, PTT–chemotherapy combination therapy offers a superior therapeutic outlook, particularly for treating refractory and drug-resistant melanomas. Dehghankhold et al. (2024) designed and synthesized a multifunctional magnetic nanosystem—Fe_3_O_4_@PDA@BSA-DOX—that integrates MRI, chemotherapy, and PTT into a single platform. In this system, Fe_3_O_4_ nanoparticles are coated with PDA to enhance photothermal conversion efficiency, further modified with BSA, and loaded with a thermally responsive azobenzene-doxorubicin (azo-DOX) prodrug for controlled release. The Fe_3_O_4_ core provides excellent T_2_-weighted imaging capability, while the PDA coating allows efficient photothermal conversion under 808 nm NIR laser irradiation, increasing the local temperature to 47 °C and thereby triggering on-demand drug release. *In vitro* studies confirmed that DOX release was both temperature- and pH-responsive. Cellular and *in vivo* evaluations demonstrated minimal toxicity, high therapeutic efficacy, and significant tumor inhibition in a melanoma-bearing mouse model. In the combination treatment group, Fe_3_O_4_@PDA@BSA-DOX coupled with NIR irradiation exhibited superior tumor inhibition compared to chemotherapy or PTT alone, with no significant adverse effects.

Additionally, recent studies have shown that IONPs possess the potential to synergize with ferroptosis and PDT to enhance anti-tumor immunotherapy (Li H. et al., 2024). IONPs encapsulating PSs can release Fe^2+^ under the acidic TME, triggering Fenton reactions that induce ferroptosis. This process activates macrophage polarization toward the tumor-suppressive M1 phenotype (Xu et al., 2025). When combined with PDT, the oxidative stress within the TME is further exacerbated, resulting in synergistic anti-tumor effects (Huang et al., 2023). Therefore, IONPs not only serve as effective MRI contrast agents but also enhance the efficacy of PDT and may act as pivotal mediators linking ferroptosis, PDT, and immunotherapy. This approach offers a promising solution to overcome the limited efficacy of PDT alone in melanoma treatment.

In summary, melanoma treatment is entering a new era of development, with combination therapy strategies demonstrating unprecedented therapeutic potential. Nanomedicine-based combination therapies not only hold promise in overcoming the high drug resistance and long-term toxicity frequently associated with conventional treatments, but also enhance the efficacy of emerging therapeutic approaches. By integrating multiple therapeutic modalities, nanomedicine-enabled combinatorial regimens can capitalize on the respective advantages of individual agents to effectively suppress tumor recurrence and distant metastasis, thereby contributing to prolonged disease-free survival and overall survival in melanoma patients.

## 5 Current clinical status and regulatory challenges

A growing body of preclinical studies has demonstrated the excellent performance of IONPs in diagnostic imaging and targeted therapy; however, their clinical translation remains relatively limited. Clinically approved or advanced IONPs formulations are mainly concentrated in the fields of MRI contrast enhancement and magnetic hyperthermia therapy. Representative products such as Ferumoxytol (Feraheme®), Feridex®, and Resovist® have obtained regulatory approval for use as MRI contrast agents or in the treatment of iron deficiency anemia and have been further explored for imaging and therapeutic monitoring of hepatocellular carcinoma, breast cancer, brain tumors, and metastatic melanoma (Coll-Font and Nguyen, 2023). For example, a clinical imaging study of Ferumoxytol in central nervous system inflammatory lesions (NCT00659776) demonstrated favorable contrast properties and safety profiles. In addition, several preclinical investigations have shown that IONP-based formulations can enhance intratumoral drug accumulation and local therapeutic efficacy through the EPR effect combined with external magnetic field guidance. Nevertheless, IONPs formulations specifically designed for melanoma remain scarce in clinical trials, most of which are still at early phase I/II validation stages. Systematic clinical data verifying their long-term safety and therapeutic efficacy are still lacking. From a regulatory and translational standpoint, the clinical implementation of IONPs faces multiple challenges. First, the characterization standards for nanomaterials are not yet unified; subtle variations in particle size distribution, surface charge, coating composition, and magnetic responsiveness can significantly influence their *in vivo* metabolism and toxicological behavior. Second, achieving batch-to-batch consistency in manufacturing and quality control remains difficult, complicating clinical evaluation and regulatory approval. Third, current drug safety assessment frameworks are primarily designed for small molecules and biologics, lacking systematic evaluation criteria for the long-term toxicity, immunogenicity, and metabolic persistence of nanomaterials. Moreover, significant discrepancies exist between the pharmacokinetic behaviors of IONPs in animal models and humans, limiting their clinical predictability. To advance the clinical translation of IONP-based theranostic platforms for melanoma, breakthroughs are needed in standardized formulation production, nano-toxicological evaluation systems, and interdisciplinary regulatory frameworks. The establishment of unified quality control standards for nanomedicines, combined with multimodal imaging-based tracking technologies and comprehensive toxicological assessment methods, will improve the reproducibility and safety of IONPs formulations, thereby accelerating their transition from laboratory research to clinical application.

## 6 Summary and outlook

Over the past decade, IONPs have made remarkable progress in melanoma diag-nosis and therapy, exhibiting unique advantages in magnetic resonance imaging, mag-netic hyperthermia, and drug delivery. However, their translation from laboratory re-search to clinical application still faces multiple challenges. Currently, only a limited number of nanomedicines have been approved by the FDA, and most IONP-related investigations remain at the preclinical stage. Key issues—including the structural sta-bility of drug–nanoparticle conjugates, targeting accuracy, optimization of particle size and surface modification, controllable drug release *in vivo*, and immunological safe-ty—require systematic resolution. Future research should focus on designing intelli-gent hybrid nanoplatforms with multi-stimuli responsiveness to magnetic, optical, pH, enzymatic, or redox cues, enabling precise and controllable drug release and multi-modal synergistic therapy. The incorporation of artificial intelligence (AI) and mul-tiscale modeling—through machine learning and molecular dynamics simulations—will allow data-driven optimization of IONPs’ size, morphology, magnetic properties, and biodistribution, thus guiding rational nanomedicine design. Furthermore, the inte-gration of radiomics with multi-omics data should be strengthened to develop person-alized therapeutic strategies based on IONPs, identify differential responses among melanoma patients with distinct molecular subtypes, and establish a closed-loop “di-agnosis–therapy–feedback” framework. On this basis, combining multimodal imaging techniques such as MRI, PET, NIR-II fluorescence, and photoacoustic imaging will enable real-time tracking and quantitative analysis of IONPs’ biodistribution, metabo-lism, and therapeutic responses, providing robust evidence for clinical translation. In addition, elucidating the mechanisms by which IONPs regulate the tumor immune mi-croenvironment remains crucial, particularly their influence on TAM polarization, dendritic cell activation, and T-cell responses. Such insights will promote the devel-opment of multidimensional therapeutic systems integrating magnetic hyperthermia, photothermal, photodynamic, and immunotherapeutic modalities. Through multidisci-plinary innovation, AI-assisted design, and systems biology integration, future studies on IONPs in melanoma theranostics are expected to evolve toward intelligent, precise, and personalized directions, establishing a solid foundation for safe, controllable, and efficient clinical translation.

## References

[B1] Amos-Tautua B. M. Songca S. P. Oluwafemi O. S. (2019). Application of porphyrins in antibacterial photodynamic therapy. Molecules 24, 2456. 10.3390/molecules24132456 31277423 PMC6650910

[B2] Anand U. Dey A. Chandel A. K. S. Sanyal R. Mishra A. Pandey D. K. (2023). Cancer chemotherapy and beyond: current status, drug candidates, associated risks and progress in targeted therapeutics. Genes. Dis. 10, 1367–1401. 10.1016/j.gendis.2022.02.007 37397557 PMC10310991

[B3] Apalla Z. Nashan D. Weller R. B. Castellsagué X. Sankella S. (2017). Skin cancer: epidemiology, disease burden, pathophysiology, diagnosis, and therapeutic approaches. Dermatol. Ther. 7 (Suppl. 1), 5–19. 10.1007/s13555-016-0165-y 28150105 PMC5289116

[B4] Bala V. M. Lampropoulou D. I. Grammatikaki S. Kouloulias V. Lagopati N. Aravantinos G. (2023). Nanoparticle-mediated hyperthermia and cytotoxicity mechanisms in cancer. Int. J. Mol. Sci. 25, 296. 10.3390/ijms25010296 38203467 PMC10779099

[B5] Baroli B. Ennas M. G. Loffredo F. Isola M. Pinna R. López-Quintela M. A. (2007). Penetration of metallic nanoparticles in human full-thickness skin. J. Investig. Dermatol. 127, 1701–1712. 10.1038/sj.jid.5700733 17380118

[B6] Beigi F. H. Jazi S. S. Shahbazi-Gahrouei D. Khaniabadi P. M. Hafezi H. Monajemi R. (2022). Iron oxide nanoparticles coated with polydopamine as a potential nano-photothermal agent for treatment of melanoma cancer: an *in vivo* study. Lasers Med. Sci. 37, 3413–3421. 10.1007/s10103-022-03599-9 35900685

[B7] Bellizzi G. Bucci O. M. (2010). On the optimal choice of the exposure conditions and the nanoparticle features in magnetic nanoparticle hyperthermia. Int. J. Hyperth. 26, 389–403. 10.3109/02656730903514685 20210609

[B8] Blanco-Andujar C. Ortega D. Southern P. Nesbitt S. A. Thanh N. T. K. Pankhurst Q. A. (2016). Real-time tracking of delayed-onset cellular apoptosis induced by intracellular magnetic hyperthermia. Nanomedicine 11, 121–136. 10.2217/nnm.15.185 26654549

[B9] Busquets M. A. Fernández J. M. Serra P. Estelrich J. (2020). Superparamagnetic nanoparticles with efficient near-infrared photothermal effect at the second biological window. Molecules 25, 5315. 10.3390/molecules25225315 33202640 PMC7696853

[B10] Cengelli F. Grzyb J. A. Montoro A. Hofmann H. Hanessian S. Juillerat-Jeanneret L. (2009). Surface-functionalized ultrasmall superparamagnetic nanoparticles as magnetic delivery vectors for camptothecin. ChemMedChem 4, 988–997. 10.1002/cmdc.200800424 19347834

[B11] Chao Y. Chen G. Liang C. Xu J. Dong Z. Han X. (2019). Iron nanoparticles for low-power local magnetic hyperthermia in combination with immune checkpoint blockade for systemic antitumor therapy. Nano Lett. 19, 4287–4296. 10.1021/acs.nanolett.9b00579 31132270

[B12] Chen Q. Zhou J. Chen Z. Luo Q. Xu J. Song G. (2019). Tumor-specific expansion of oxidative stress by glutathione depletion and use of a fenton nanoagent for enhanced chemodynamic therapy. ACS Appl. Mat. Interfaces 11, 30551–30565. 10.1021/acsami.9b09323 31397998

[B13] Chen F. Li T. Zhang H. Saeed M. Liu X. Huang L. (2023a). Acid-ionizable iron nanoadjuvant augments STING activation for personalized vaccination immunotherapy of cancer. Adv. Mat. 35, e2209910. 10.1002/adma.202209910 36576344

[B14] Chen Y. Yang Y. Du S. Ren J. Jiang H. Zhang L. (2023b). Mitochondria-targeting upconversion nanoparticles@MOF for multiple-enhanced photodynamic therapy in hypoxic tumor. ACS Appl. Mat. Interfaces 15, 35884–35894. 10.1021/acsami.3c05447 37487181

[B15] Chiang C. S. Lin Y. J. Lee R. Lai Y. H. Cheng H. W. Hsieh C. H. (2018). Combination of fucoidan-based magnetic nanoparticles and immunomodulators enhances tumour-localized immunotherapy. Nat. Nanotechnol. 13, 746–754. 10.1038/s41565-018-0146-7 29760523

[B16] Chomoucka J. Drbohlavova J. Huska D. Adam V. Kizek R. Hubalek J. (2010). Magnetic nanoparticles and targeted drug delivering. Pharmacol. Res. 62, 144–149. 10.1016/j.phrs.2010.01.014 20149874

[B17] Coll-Font J. Nguyen C. (2023). Editorial for “IOP Injection, a novel superparamagnetic iron oxide particle MRI contrast agent for the detection of hepatocellular carcinoma: a phase II clinical trial.”. J. Magn. Reson. Imaging 58, 1189–1190. 10.1002/jmri.28657 36820512

[B18] Connord V. Clerc P. Hallali N. El Hajj Diab D. Fourmy D. Gigoux V. (2015). Real-time analysis of magnetic hyperthermia experiments on living cells under a confocal microscope. Small 11, 2437–2445. 10.1002/smll.201402669 25644392

[B19] Corte-Real M. Veiga F. Paiva-Santos A. C. Pires P. C. (2024). Improving skin cancer treatment by dual drug co-encapsulation into liposomal systems—an integrated approach towards anticancer synergism and targeted delivery. Pharmaceutics 16, 1200. 10.3390/pharmaceutics16091200 39339235 PMC11434718

[B20] Cruz N. Pinho J. O. Soveral G. Ascensão L. Matela N. Reis C. (2020). A novel hybrid nanosystem integrating cytotoxic and magnetic properties as a tool to potentiate melanoma therapy. Nanomaterials 10, 693. 10.3390/nano10040693 32268611 PMC7221742

[B21] Daya R. Xu C. Nguyen N. Y. T. Liu H. H. (2022). Angiogenic hyaluronic acid hydrogels with curcumin-coated magnetic nanoparticles for tissue repair. ACS Appl. Mat. Interfaces 14, 11051–11067. 10.1021/acsami.1c19889 35199989

[B22] de Carvalho Lima E. N. Barros Martins G. L. Diaz R. S. Schechter M. Piqueira J. R. C. Justo J. F. (2022). Effects of carbon nanomaterials and Aloe vera on melanomas—where are we? Recent updates. Pharmaceutics 14, 2004. 10.3390/pharmaceutics14102004 36297440 PMC9607275

[B23] Dehghankhold M. Ahmadi F. Nezafat N. Abedi M. Iranpour P. Dehghanian A. (2024). A versatile theranostic magnetic polydopamine iron oxide NIR laser-responsive nanosystem containing doxorubicin for chemo-photothermal therapy of melanoma. Biomater. Adv. 159, 213797. 10.1016/j.bioadv.2024.213797 38368693

[B24] Deng L. Liu M. Sheng D. Luo Y. Wang D. Yu X. (2021). Low-intensity focused ultrasound-augmented cascade chemodynamic therapy *via* boosting ROS generation. Biomaterials 271, 120710. 10.1016/j.biomaterials.2021.120710 33610047

[B25] Dhillon S. K. Porter S. L. Rizk N. Sheng Y. McKaig T. Burnett K. (2020). Rose Bengal-amphiphilic peptide conjugate for enhanced photodynamic therapy of malignant melanoma. J. Med. Chem. 63, 1328–1336. 10.1021/acs.jmedchem.9b01802 31940202

[B26] Domenech M. Marrero-Berrios I. Torres-Lugo M. Rinaldi C. (2013). Lysosomal membrane permeabilization by targeted magnetic nanoparticles in alternating magnetic fields. ACS Nano 7, 5091–5101. 10.1021/nn4007048 23705969

[B27] Domingues B. Lopes J. M. Soares P. Pópulo H. (2018). Melanoma treatment in review. Immuno Targets Ther. 7, 35–49. 10.2147/ITT.S134842 29922629 PMC5995433

[B28] Duan Y. Wu X. Gong Z. Guo Q. Kong Y. (2022). Pathological impact and medical applications of electromagnetic field on melanoma: a focused review. Front. Oncol. 12, 857068. 10.3389/fonc.2022.857068 35936711 PMC9355252

[B29] Duan X. Wang P. He L. He Z. Wang S. Yang F. (2024). Peptide-functionalized inorganic oxide nanomaterials for solid cancer imaging and therapy. Adv. Mat. 36, e2311548. 10.1002/adma.202311548 38333964

[B30] Duval K. E. A. Vernice N. A. Wagner R. J. Fiering S. N. Petryk J. D. Lowry G. J. (2019). Immunogenetic effects of low dose (CEM43 30) magnetic nanoparticle hyperthermia and radiation in melanoma cells. Int. J. Hyperth. 36 (Suppl. 1), 37–46. 10.1080/02656736.2019.1627433 31795829 PMC6943912

[B31] Ezike T. C. Okpala U. S. Onoja U. L. Nwike C. P. Ezeako E. C. Okpara O. J. (2023). Advances in drug delivery systems, challenges and future directions. Heliyon 9, e17488. 10.1016/j.heliyon.2023.e17488 37416680 PMC10320272

[B32] Fu Y. Jang M. S. Liu C. Li Y. Lee J. H. Yang H. Y. (2023). Oxygen-generating organic/inorganic self-assembled nanocolloids for tumor-activated dual-model imaging-guided photodynamic therapy. ACS Appl. Mat. Interfaces 15, 36013–36024. 10.1021/acsami.3c07008 37478563

[B33] Gao J. Gu H. Xu B. (2009). Multifunctional magnetic nanoparticles: design, synthesis, and biomedical applications. Acc. Chem. Res. 42, 1097–1107. 10.1021/ar9000026 19476332

[B34] García-Soriano D. Milán-Rois P. Lafuente-Gómez N. Rodríguez-Díaz C. Navío C. Somoza Á. (2024). Multicore iron oxide nanoparticles for magnetic hyperthermia and combination therapy against cancer cells. J. Colloid Interface Sci. 670, 73–85. 10.1016/j.jcis.2024.05.046 38759270

[B35] Gladfelter P. Darwish N. H. E. Mousa S. A. (2017). Current status and future direction in the management of malignant melanoma. Melanoma Res. 27, 403–410. 10.1097/CMR.0000000000000379 28800028

[B36] Gomes A. A. Valverde T. M. Machado V. de O. do Nascimento da Silva E. Fagundes D. A. Oliveira F. de P. (2023). Heating capacity and biocompatibility of hybrid nanoparticles for magnetic hyperthermia treatment. Int. J. Mol. Sci. 25, 493. 10.3390/ijms25010493 38203662 PMC10779024

[B37] Gray-Schopfer V. Wellbrock C. Marais R. (2007). Melanoma biology and new targeted therapy. Nature 445, 851–857. 10.1038/nature05661 17314971

[B38] Grossman C. E. Pickup S. Durham A. Wileyto E. P. Putt M. E. Busch T. M. (2011). Photodynamic therapy of disseminated non-small cell lung carcinoma in a murine model. Lasers Surg. Med. 43, 663–675. 10.1002/lsm.21102 22057494 PMC3676899

[B39] Haase T. Ludwig A. Stach A. Mohtashamdolatshahi A. Hauptmann R. Mundhenk L. (2024). Repeated injection of very small superparamagnetic iron oxide particles (VSOPs) in murine atherosclerosis: a safety study. Nanomater. (Basel) 14, 773. 10.3390/nano14090773 38727367 PMC11085881

[B40] Heras-Murillo I. Adán-Barrientos I. Galán M. Wculek S. K. Sancho D. (2024). Dendritic cells as orchestrators of anticancer immunity and immunotherapy. Nat. Rev. Clin. Oncol. 21, 257–277. 10.1038/s41571-024-00859-1 38326563

[B41] Hou Y. Zhang P. Wang D. Liu J. Rao W. (2020). Liquid metal hybrid platform-mediated ice-fire dual noninvasive conformable melanoma therapy. ACS Appl. Mat. Interfaces 12, 27984–27993. 10.1021/acsami.0c06023 32463667

[B42] Hou Y. Sun X. Dou M. Lu C. Liu J. Rao W. (2021). Cellulose nanocrystals facilitate needle-like ice crystal growth and modulate molecular targeted ice crystal nucleation. Nano Lett. 21, 4868–4877. 10.1021/acs.nanolett.1c00514 33819045

[B43] Hu S. H. Liu T. Y. Liu D. M. Chen S. Y. (2007). Controlled pulsatile drug release from a ferrogel by a high-frequency magnetic field. Macromolecules 40, 6786–6788. 10.1021/ma0707584

[B44] Hu T. Wang Z. Shen W. Liang R. Yan D. Wei M. (2021). Recent advances in innovative strategies for enhanced cancer photodynamic therapy. Theranostics 11, 3278–3300. 10.7150/thno.54227 33537087 PMC7847668

[B45] Huang Y. Li X. Zhang Z. Xiong L. Wang Y. Wen Y. (2023). Photodynamic therapy combined with ferroptosis is a synergistic antitumor therapy strategy. Cancers. 15, 5043. 10.3390/cancers15205043 37894410 PMC10604985

[B46] Ikeda-Imamuku M. Wang L. L. W. Rodrigues D. Shaha S. Zhao Z. Mitragotri S. (2022). Strategies to improve the EPR effect: a mechanistic perspective and clinical translation. J. Control. Release 345, 512–536. 10.1016/j.jconrel.2022.03.043 35337939

[B47] Ilg P. Kröger M. (2020). Dynamics of interacting magnetic nanoparticles: effective behavior from competition between Brownian and Néel relaxation. Phys. Chem. Chem. Phys. 22, 22244–22259. 10.1039/D0CP04377J 33001111

[B48] Jacinto C. Javed Y. Lavorato G. Tarraga W. A. Conde B. I. C. Orozco J. M. (2025). Biotransformation and biological fate of magnetic iron oxide nanoparticles for biomedical research and clinical applications. Nanoscale Adv. 7, 2818–2886. 10.1039/D5NA00195A 40255989 PMC12004083

[B49] Janaszewska A. Studzian M. Petersen J. F. Ficker M. Christensen J. B. Klajnert-Maculewicz B. (2015). PAMAM dendrimer with 4-carbomethoxypyrrolidone—in vitro assessment of neurotoxicity. Nanomedicine 11, 409–411. 10.1016/j.nano.2014.09.011 25461280

[B50] Jeon S. Subbiah R. Bonaedy T. Van S. Park K. Yun K. (2018). Surface functionalized magnetic nanoparticles shift cell behavior with on/off magnetic fields. J. Cell. Physiol. 233, 1168–1178. 10.1002/jcp.25980 28464242

[B51] Jiang H. Fu H. Guo Y. Hu P. Shi J. (2022). Evoking tumor-associated macrophages by mitochondria-targeted magnetothermal immunogenic cell death for cancer immunotherapy. Biomaterials 289, 121799. 10.1016/j.biomaterials.2022.121799 36152515

[B52] Johnson D. B. Pollack M. H. Sosman J. A. (2016). Emerging targeted therapies for melanoma. Expert Opin. Emerg. Drugs 21, 195–207. 10.1080/14728214.2016.1184644 27148822

[B53] Kim J. Cho H. Lim D. K. Joo M. K. Kim K. (2023). Perspectives for improving the tumor targeting of nanomedicine *via* the EPR effect in clinical tumors. Int. J. Mol. Sci. 24, 10082. 10.3390/ijms241210082 37373227 PMC10298311

[B54] Kladko D. V. Falchevskaya A. S. Serov N. S. Prilepskii A. Y. (2021). Nanomaterial shape influence on cell behavior. Int. J. Mol. Sci. 22, 5266. 10.3390/ijms22105266 34067696 PMC8156540

[B55] Krawczyk P. M. Eppink B. Essers J. Stap J. Rodermond H. Odijk H. (2011). Mild hyperthermia inhibits homologous recombination, induces BRCA2 degradation, and sensitizes cancer cells to poly (ADP-ribose) polymerase-1 inhibition. Proc. Natl. Acad. Sci. U.S.A. 108, 9851–9856. 10.1073/pnas.1101053108 21555554 PMC3116433

[B56] Kwak K. Yu B. Lewandowski R. J. Kim D.-H. (2022). Recent progress in cryoablation cancer therapy and nanoparticles mediated cryoablation. Theranostics 12, 2175–2204. 10.7150/thno.67530 35265206 PMC8899563

[B57] Kwiatkowski S. Knap B. Przystupski D. Saczko J. Kędzierska E. Knap-Czop K. (2018). Photodynamic therapy – mechanisms, photosensitizers and combinations. Biomed. Pharmacother. 106, 1098–1107. 10.1016/j.biopha.2018.07.049 30119176

[B58] Li X. Zhao D. Zhang F. (2013). Multifunctional upconversion-magnetic hybrid nanostructured materials: synthesis and bioapplications. Theranostics 3, 292–305. 10.7150/thno.5289 23650477 PMC3645056

[B59] Li K. Nejadnik H. Daldrup-Link H. E. (2017). Next-generation superparamagnetic iron oxide nanoparticles for cancer theranostics. Drug Discov. Today 22, 1421–1429. 10.1016/j.drudis.2017.04.008 28454771 PMC5610947

[B60] Li M. Bu W. Ren J. Li J. Deng L. Gao M. (2018). Enhanced synergism of thermo-chemotherapy for liver cancer with magnetothermally responsive nanocarriers. Theranostics 8, 693–709. 10.7150/thno.21297 29344299 PMC5771086

[B61] Li L. Zhang X. Zhou J. Zhang L. Xue J. Tao W. (2022). Non-invasive thermal therapy for tissue engineering and regenerative medicine. Small 18, e2107705. 10.1002/smll.202107705 35475541

[B62] Li B. Hao Y. He H. Fan Y. Ren B. Peng X. (2024a). CD47-SIRPα blockade sensitizes head and neck squamous cell carcinoma to cetuximab by enhancing macrophage adhesion to cancer cells. Cancer Res. 84, 3189–3206. 10.1158/0008-5472.CAN-24-0176 38959336

[B63] Li M. Li Y. Zheng J. Ma Z. Zhang J. Wu H. (2024b). Ultrasound-responsive nanocarriers with siRNA and Fe_3_O_4_ regulate macrophage polarization and phagocytosis for augmented non-small cell lung cancer immunotherapy. J. Nanobiotechnol. 22, 605. 10.1186/s12951-024-02883-w 39375761 PMC11460142

[B64] Li H. Dou Y. Yang H. Xing H. Zhu C. Wang T. (2024c). Ce6-modified Fe ions-doped carbon dots as multifunctional nanoplatform for ferroptosis and photodynamic synergistic therapy of melanoma. J. Nanobiotechnol. 22, 100. 10.1186/s12951-024-02346-2 38462597 PMC10924998

[B65] Liu J. Li B. Li L. Ming X. Xu Z. P. (2024). Advances in nanomaterials for immunotherapeutic improvement of cancer chemotherapy. Small 20, e2403024. 10.1002/smll.202403024 38773882

[B66] Liu H. Zhen Z. Chen F. Chen J. Chen W. (2025). Advancements in iron oxide nanoparticles for multimodal imaging and tumor theranostics. Curr. Med. Chem. 32, 301–321. 10.2174/0109298673301359240705063544 39005127

[B67] Liu-Smith F. Jia J. Zheng Y. (2017). UV-induced molecular signaling differences in melanoma and non-melanoma skin cancer. Adv. Exp. Med. Biol. 996, 27–40. 10.1007/978-3-319-56017-5_3 29124688

[B68] Long G. V. Menzies A. M. Scolyer R. A. (2023). Neoadjuvant checkpoint immunotherapy and melanoma: the time is now. J. Clin. Oncol. 41, 3236–3248. 10.1200/JCO.22.02575 37104746

[B69] Long L. Xinwei K. Yi H. Zhu L. Lau P. Li X. (2025). Alterations in PD-L1 succinylation shape anti-tumor immune responses in melanoma. Nat. Genet. 57, 680–693. 10.1038/s41588-025-02077-6 40069506 PMC11906371

[B70] Lopez S. Hallali N. Lalatonne Y. Hillion A. Antunes J. C. Serhan N. (2022). Magneto-mechanical destruction of cancer-associated fibroblasts using ultra-small iron oxide nanoparticles and low frequency rotating magnetic fields. Nanoscale Adv. 4, 421–436. 10.1039/d1na00474c 36132704 PMC9417452

[B71] Luke J. J. Schwartz G. K. (2013). Chemotherapy in the management of advanced cutaneous malignant melanoma. Clin. Dermatol. 31, 290–297. 10.1016/j.clindermatol.2012.08.016 23608448 PMC3709980

[B72] Luo L. Iqbal M. Z. Liu C. Xing J. Akakuru O. U. Fang Q. (2019). Engineered nano-immunopotentiators efficiently promote cancer immunotherapy for inhibiting and preventing lung metastasis of melanoma. Biomaterials 223, 119464. 10.1016/j.biomaterials.2019.119464 31525691

[B73] Malhotra N. Lee J. S. Liman R. A. D. Ruallo J. M. S. Villaflores O. B. Ger T. R. (2020). Potential toxicity of iron oxide magnetic nanoparticles: a review. Molecules 25, 3159. 10.3390/molecules25143159 32664325 PMC7397295

[B74] Marzi M. Osanloo M. Vakil M. K. Mansoori Y. Ghasemian A. Dehghan A. (2022). Applications of metallic nanoparticles in the skin cancer treatment. Biomed. Res. Int. 2022, 2346941. 10.1155/2022/2346941 36420097 PMC9678447

[B75] Montaseri H. Kruger C. A. Abrahamse H. (2021). Inorganic nanoparticles applied for active targeted photodynamic therapy of breast cancer. Pharmaceutics 13, 296. 10.3390/pharmaceutics13030296 33668307 PMC7996317

[B76] My C. F Z. Sp G. We G. (2024). Dendritic cell subsets and implications for cancer immunotherapy. Front. Immunol. 15, 1393451. 10.3389/fimmu.2024.1393451 38903502 PMC11188312

[B77] Naidoo C. Kruger C. A. Abrahamse H. (2018). Photodynamic therapy for metastatic melanoma treatment: a review. Technol. Cancer Res. Treat. 17, 1533033818791795. 10.1177/1533033818791795 30099929 PMC6090489

[B78] Nascimento C. S. Alves É. A. R. De Melo C. P. Corrêa-Oliveira R. Calzavara-Silva C. E. (2021). Immunotherapy for cancer: effects of iron oxide nanoparticles on polarization of tumor-associated macrophages. Nanomedicine 16, 2633–2650. 10.2217/nnm-2021-0255 34854309

[B79] Nguyen K. Hignett E. Khachemoune A. (2020). Current and emerging treatment options for metastatic melanoma: a focused review. Dermatol. Online J. 26. 10.5070/D3267049551 32898395

[B80] Nistorescu S. Udrea A. M. Badea M. A. Lungu I. Boni M. Tozar T. (2021). Low blue dose photodynamic therapy with porphyrin-iron oxide nanoparticles complexes: *in vitro* study on human melanoma cells. Pharmaceutics 13, 2130. 10.3390/pharmaceutics13122130 34959411 PMC8705854

[B81] Nkune N. W. Kruger C. A. Abrahamse H. (2021). Possible enhancement of photodynamic therapy (PDT) colorectal cancer treatment when combined with cannabidiol. Anti-Cancer Agents Med. Chem. 21, 137–148. 10.2174/1871520620666200415102321 32294046

[B82] Opolka-Hoffmann E. Jordan G. Otteneder M. Kieferle R. Lechmann M. Winter G. (2021). The impact of immunogenicity on therapeutic antibody pharmacokinetics: a preclinical evaluation of the effect of immune complex formation and antibody effector function on clearance. mAbs 13, 1995929. 10.1080/19420862.2021.1995929 34763611 PMC8726625

[B83] Osorio J. C. Smith P. Knorr D. A. Ravetch J. V. (2023). The antitumor activities of anti-CD47 antibodies require Fc-FcγR interactions. Cancer Cell. 41, 2051–2065.e6. 10.1016/j.ccell.2023.10.007 37977147 PMC10842210

[B84] Paez-Muñoz J. M. Gámez F. Fernández-Afonso Y. Gallardo R. Pernia Leal M. Gutiérrez L. (2023). Optimization of iron oxide nanoparticles for MRI-guided magnetic hyperthermia tumor therapy: reassessing the role of shape in their magnetocaloric effect. J. Mat. Chem. B 11, 11110–11120. 10.1039/d3tb01821k 37947078

[B85] Pandesh S. Haghjooy Javanmard S. Shakeri-Zadeh A. Shokrani P. (2021). Targeted photothermal therapy of melanoma in C57BL/6 mice using Fe_3_O_4_@Au core–shell nanoparticles and near-infrared laser. J. Biomed. Phys. Eng. 11, 29–38. 10.31661/jbpe.v0i0.736 33564637 PMC7859370

[B86] Patrick P. S. Stuckey D. J. Zhu H. Kalber T. L. Iftikhar H. Southern P. (2024). Improved tumour delivery of iron oxide nanoparticles for magnetic hyperthermia therapy of melanoma *via* ultrasound guidance and ^111^In SPECT quantification. Nanoscale 16, 19715–19729. 10.1039/d4nr00240g 39044561 PMC11488578

[B87] Pojo M. Cerqueira S. R. Mota T. Xavier-Magalhães A. Ribeiro-Samy S. Mano J. F. (2013). *In vitro* evaluation of the cytotoxicity and cellular uptake of CMCht/PAMAM dendrimer nanoparticles by glioblastoma cell models. J. Nanopart. Res. 15, 1621. 10.1007/s11051-013-1621-6

[B88] Przygoda M. Bartusik-Aebisher D. Dynarowicz K. Cieślar G. Kawczyk-Krupka A. Aebisher D. (2023). Cellular mechanisms of singlet oxygen in photodynamic therapy. Int. J. Mol. Sci. 24, 16890. 10.3390/ijms242316890 38069213 PMC10706571

[B89] Rao Y. F. Chen W. Liang X. G. Huang Y. Miao J. Liu L. (2015). Epirubicin-loaded superparamagnetic iron-oxide nanoparticles for transdermal delivery: cancer therapy by circumventing the skin barrier. Small 11, 239–247. 10.1002/smll.201400775 24925046

[B90] Rao L. Zhao S. K. Wen C. Tian R. Lin L. Cai B. (2020). Activating macrophage-mediated cancer immunotherapy by genetically edited nanoparticles. Adv. Mat. 32, e2004853. 10.1002/adma.202004853 33089578 PMC7686299

[B91] Ratajczak F. Prajwos E. Bielas R. Józefczak A. (2025). Controlled localization of ultrasound heating using magnetic nanoparticle assemblies. Ultrason. Sonochem. 121, 107567. 10.1016/j.ultsonch.2025.107567 40945042 PMC12504973

[B92] Rego G. N. A. Nucci M. P. Mamani J. B. Oliveira F. A. Marti L. C. Filgueiras I. S. (2020). Therapeutic efficiency of multiple applications of magnetic hyperthermia technique in glioblastoma using aminosilane coated iron oxide nanoparticles: *in vitro* and *in vivo* study. Int. J. Mol. Sci. 21, 958. 10.3390/ijms21030958 32023985 PMC7038138

[B93] Repasky E. A. Evans S. S. Dewhirst M. W. (2013). Temperature matters! and why it should matter to tumor immunologists. Cancer Immunol. Res. 1, 210–216. 10.1158/2326-6066.CIR-13-0118 24490177 PMC3904378

[B94] Roelands J. van der Ploeg M. Ijsselsteijn M. E. Dang H. Boonstra J. J. Hardwick J. C. H. (2023). Transcriptomic and immunophenotypic profiling reveals molecular and immunological hallmarks of colorectal cancer tumourigenesis. Gut 72, 1326–1339. 10.1136/gutjnl-2022-327608 36442992 PMC10314051

[B95] Salmanian G. Hassanzadeh-Tabrizi S. A. Koupaei N. (2021). Magnetic chitosan nanocomposites for simultaneous hyperthermia and drug delivery applications: a review. Int. J. Biol. Macromol. 184, 618–635. 10.1016/j.ijbiomac.2021.06.108 34166696

[B96] Serrano-Puebla A. Boya P. (2018). Lysosomal membrane permeabilization as a cell death mechanism in cancer cells. Biochem. Soc. Trans. 46, 207–215. 10.1042/BST20170130 29472365

[B97] Shadbad M. A. Hajiasgharzadeh K. Derakhshani A. Silvestris N. Baghbanzadeh A. Racanelli V. (2021). From melanoma development to RNA-modified dendritic cell vaccines: highlighting the lessons from the past. Front. Immunol. 12, 623639. 10.3389/fimmu.2021.623639 33692796 PMC7937699

[B98] Shan Z. Liu F. (2025). Opportunities, obstacles and challenges of nano-immunotherapy in melanoma. Front. Immunol. 16, 1611423. 10.3389/fimmu.2025.1611423 40861441 PMC12370749

[B99] Shapouri-Moghaddam A. Mohammadian S. Vazini H. Taghadosi M. Esmaeili S.-A. Mardani F. (2018). Macrophage plasticity, polarization, and function in health and dis-ease. Cell. Physiol. 233, 6425–6440. 10.1002/jcp.26429 29319160 PMC13482560

[B100] Shi Y. Van der Meel R. Chen X. Lammers T. (2020). The EPR effect and beyond: strategies to improve tumor targeting and cancer nanomedicine treatment efficacy. Theranostics 10, 7921–7924. 10.7150/thno.49577 32685029 PMC7359085

[B101] Shubayev V. I. Pisanic T. R. Jin S. (2009). Magnetic nanoparticles for theragnostics. Adv. Drug Deliv. Rev. 61, 467–477. 10.1016/j.addr.2009.03.007 19389434 PMC2700776

[B102] Siddiqui M. A. Wahab R. Saquib Q. Ahmad J. Farshori N. N. Al-Sheddi E. S. (2023). Iron oxide nanoparticles induced cytotoxicity, oxidative stress, cell cycle arrest, and DNA damage in human umbilical vein endothelial cells. J. Trace Elem. Med. Biol. 80, 127302. 10.1016/j.jtemb.2023.127302 37734210

[B103] Singh P. Pandit S. Balusamy S. R. Madhusudanan M. Singh H. Amsath H. H. M. (2025). Advanced nanomaterials for cancer therapy: gold, silver, and iron oxide nanoparticles in oncological applications. Adv. Healthc. Mat. 14, e2403059. 10.1002/adhm.202403059 39501968 PMC11804848

[B104] Solar P. González G. Vilos C. Herrera N. Juica N. Moreno M. (2015). Multifunctional polymeric nanoparticles doubly loaded with SPION and ceftiofur retain their physical and biological properties. J. Nanobiotechnol. 13, 14. 10.1186/s12951-015-0077-5 25886018 PMC4334767

[B105] Song Q. Zhang G. Wang B. Cao G. Li D. Wang Y. (2021). Reinforcing the combinational immuno-oncotherapy of switching “cold” tumor to “hot” by responsive penetrating nano-gels. ACS Appl. Mat. Interfaces 13, 36824–36838. 10.1021/acsami.1c08201 34314148

[B106] Souiade L. Domingo-Diez J. Alcaide C. Gámez B. Gámez L. Ramos M. (2023). Improving the efficacy of magnetic nanoparticle-mediated hyperthermia using trapezoidal pulsed electromagnetic fields as an *in vitro* anticancer treatment in melanoma and glioblastoma multiforme cell lines. Int. J. Mol. Sci. 24, 15933. 10.3390/ijms242115933 37958913 PMC10648011

[B107] Sowmya S. V. Augustine D. Hosmani J. Pagnoni F. Reda R. Testarelli L. (2024). Nanoparticle-based biomolecules in cancer diagnosis, therapy, drug delivery and prognosis. Front. Dent. Med. 5, 1482166. 10.3389/fdmed.2024.1482166 39917652 PMC11797830

[B108] Suh M. Park J. Y. Ko G. B. Kim J. Y. Hwang D. W. Rees L. (2024). Optimization of micelle-encapsulated extremely small sized iron oxide nanoparticles as a T1 contrast imaging agent: biodistribution and safety profile. J. Nanobiotechnol. 22, 419. 10.1186/s12951-024-02699-8 39014410 PMC11253436

[B109] Sun L. Liu H. Ye Y. Lei Y. Islam R. Tan S. (2023). Smart nanoparticles for cancer therapy. Signal Transduct. Target. Ther. 8, 418. 10.1038/s41392-023-01642-x 37919282 PMC10622502

[B110] Switzer B. Puzanov I. Skitzki J. J. Hamad L. Ernstoff M. S. (2022). Managing metastatic melanoma in 2022: a clinical review. JCO Oncol. Pract. 18, 335–351. 10.1200/OP.21.00686 35133862 PMC9810138

[B111] Szebeni J. (2024). Evaluation of the acute anaphylactoid reactogenicity of nanoparticle-containing medicines and vaccines using the porcine CARPA model. Methods Mol. Biol. 2789, 229–243. 10.1007/978-1-0716-3786-9_23 38507008

[B112] Tamura Y. Ito A. Wakamatsu K. Kamiya T. Torigoe T. Honda H. (2022). Immunomodulation of melanoma by chemo-thermo-immunotherapy using conjugates of melanogenesis substrate NPrCAP and magnetite nanoparticles: a review. Int. J. Mol. Sci. 23, 6457. 10.3390/ijms23126457 35742905 PMC9223671

[B113] Tang J. Q. Hou X. Y. Yang C. S. Li Y. X. Xin Y. Guo W. W. (2017). Recent developments in nanomedicine for melanoma treatment. Int. J. Cancer 141, 646–653. 10.1002/ijc.30708 28340496

[B114] Tang L. Yin Y. Cao Y. Fu C. Liu H. Feng J. (2023). Extracellular vesicles-derived hybrid nanoplatforms for amplified CD47 blockade-based cancer immunotherapy. Adv. Mat. 35, e2303835. 10.1002/adma.202303835 37384818

[B115] Toderascu L. I. Sima L. E. Orobeti S. Florian P. E. Icriverzi M. Maraloiu V.-A. (2023). Synthesis and anti-melanoma activity of L-cysteine-coated iron oxide nanoparticles loaded with doxorubicin. Nanomaterials 13, 621. 10.3390/nano13040621 36838989 PMC9966685

[B116] Vilas-Boas V. Carvalho F. Espiña B. (2020). Magnetic hyperthermia for cancer treatment: main parameters affecting the outcome of *in vitro* and *in vivo* studies. Molecules 25, 2874. 10.3390/molecules25122874 32580417 PMC7362219

[B117] Vines J. B. Yoon J.-H. Ryu N.-E. Lim D.-J. Park H. (2019). Gold nanoparticles for photothermal cancer therapy. Front. Chem. 7, 167. 10.3389/fchem.2019.00167 31024882 PMC6460051

[B118] Wang J. Wang Y. Jiang X. (2024a). Targeting anticancer immunity in melanoma tumour microenvironment: unleashing the potential of adjuvants, drugs, and phytochemicals. J. Drug Target 32, 1052–1072. 10.1080/1061186X.2024.2384071 39041142

[B119] Wang K. Coutifaris P. Brocks D. Wang G. Azar T. Solis S. (2024b). Combination anti-PD-1 and anti-CTLA-4 therapy generates waves of clonal responses that include progenitor-exhausted CD8^+^ T cells. Cancer Cell. 42, 1582–1597.e10. 10.1016/j.ccell.2024.08.007 39214097 PMC11387127

[B120] Wang S. Peng P. Wang J. Zhang Z. Liu P. Xu L. X. (2024c). Cryo-thermal therapy reshaped the tumor immune microenvironment to enhance the efficacy of adoptive T cell therapy. Cancer Immunol. Immunother. 74, 21. 10.1007/s00262-024-03884-2 39535552 PMC11561218

[B121] Wu J. (2021). The enhanced permeability and retention (EPR) effect: the significance of the concept and methods to enhance its application. J. Pers. Med. 11, 771. 10.3390/jpm11080771 34442415 PMC8402171

[B122] Wu J. (2022). Selective enhancing blood flow in solid tumor tissue is the key for achieving satisfactory delivery and therapeutic outcome of nanodrugs *via* the EPR effect. J. Pers. Med. 12, 1802. 10.3390/jpm12111802 36579542 PMC9697866

[B123] Wu X. Suo Y. Shi H. Liu R. Wu F. Wang T. (2020). Deep-tissue photothermal therapy using laser illumination at NIR-IIa window. Nano-Micro Lett. 12, 38. 10.1007/s40820-020-0378-6 34138257 PMC7770864

[B124] Xu W. Guan G. Yue R. Dong Z. Lei L. Kang H. (2025). Chemical design of magnetic nanomaterials for imaging and ferroptosis-based cancer therapy. Chem. Rev. 125, 1897–1961. 10.1021/acs.chemrev.4c00546 39951340

[B125] Yang Y. Liu Y. Song L. Cui X. Zhou J. Jin G. (2023). Iron oxide nanoparticle-based nanocomposites in biomedical application. Trends Biotechnol. 41, 1471–1487. 10.1016/j.tibtech.2023.06.001 37407395

[B126] Yang C. Li S. Wang L. (2025). Engineered iron oxide nanoplatforms: reprogramming immunosuppressive niches for precision cancer theranostics. Mol. Cancer 24, 225. 10.1186/s12943-025-02443-2 40887613 PMC12400778

[B127] Ye P. Kong Y. Chen X. Li W. Liu D. Xie Y. (2017). Fe_3_O_4_ nanoparticles and cryoablation enhance ice crystal formation to improve the efficiency of killing breast cancer cells. Oncotarget 8, 11389–11399. 10.18632/oncotarget.13859 27974703 PMC5355273

[B128] Yi J. Liu L. Gao W. Zeng J. Chen Y. Pang E. (2024). Advances and perspectives in phototherapy-based combination therapy for cancer treatment. J. Mat. Chem. B 12, 6285–6304. 10.1039/d4tb00483c 38895829

[B129] Yoncheva K. Merino M. Shenol A. Daskalov N. T. Petkov P. S. Vayssilov G. N. (2019). Optimization and *in-vitro*/*in-vivo* evaluation of doxorubicin-loaded chitosan-alginate nanoparticles using a melanoma mouse model. Int. J. Pharm. 556, 1–8. 10.1016/j.ijpharm.2018.11.070 30529664

[B130] Yu Z. Gan Z. Wu W. Sun X. Cheng X. Chen C. (2024). Photothermal-triggered extracellular matrix clearance and dendritic cell maturation for enhanced osteosarcoma immunotherapy. ACS Appl. Mat. Interfaces 16, 67225–67234. 10.1021/acsami.4c12532 39589815

[B131] Zeng Y. Gao Y. He L. Ge W. Wang X. Ma T. (2024). Smart delivery vehicles for cancer: categories, unique roles and therapeutic strategies. Nanoscale Adv. 6, 4275–4308. 10.1039/d4na00285g 39170969 PMC11334973

[B132] Zhang F. Zhao L. Wang S. Yang J. Lu G. Luo N. (2018). Construction of a biomimetic magnetosome and its application as a siRNA carrier for high-performance anticancer therapy. Adv. Funct. Mat. 28, 1703326. 10.1002/adfm.201703326

[B133] Zhao Y. Zhao X. Wang X. Ma Z. Yan J. Li S. (2025). Polyphenol-mediated assembly of toll-like receptor 7/8 agonist nanoparticles for effective tumor immunotherapy. Acta Biomater. 193, 417–428. 10.1016/j.actbio.2024.12.060 39746528

[B134] Zhi D. Yang T. Yang J. Fu S. Zhang S. (2020). Targeting strategies for superparamagnetic iron oxide nanoparticles in cancer therapy. Acta Biomater. 102, 13–34. 10.1016/j.actbio.2019.11.027 31759124

